# Estimation and probabilistic projection of levels and trends in the sex ratio at birth in seven provinces of Nepal from 1980 to 2050: a Bayesian modeling approach

**DOI:** 10.1186/s12889-022-12693-0

**Published:** 2022-02-19

**Authors:** Fengqing Chao, Samir KC, Hernando Ombao

**Affiliations:** 1 Statistics Program, Computer, Electrical and Mathematical Sciences and Engineering Division, King Abdullah University of Science and Technology, Thuwal, 23955-6900 Saudi Arabia; 2 Asian Demographic Research Institute, Shanghai University, Shangda Road, Shanghai, 200444 China; 3 Wittgenstein Centre for Demography and Global Human Capital (IIASA, OeAW, University of Vienna), International Institute for Applied Systems Analysis, Laxenburg, Austria

**Keywords:** Sex ratio at birth, Sex-selective abortion, Son preference, Bayesian hierarchical model, Nepal, Probabilistic projection, Subnational modeling

## Abstract

**Background:**

The sex ratio at birth (SRB; ratio of male to female births) in Nepal has been reported around the normal level on the national level. However, the national SRB could mask the disparity within the country. Given the demographic and cultural heterogeneities in Nepal, it is crucial to model Nepal SRB on the subnational level. Prior studies on subnational SRB in Nepal are mostly based on reporting observed values from surveys and census, and no study has provided probabilistic projections. We aim to estimate and project SRB for the seven provinces of Nepal from 1980 to 2050 using a Bayesian modeling approach.

**Methods:**

We compiled an extensive database on provincial SRB of Nepal, consisting 2001, 2006, 2011, and 2016 Nepal Demographic and Health Surveys and 2011 Census. We adopted a Bayesian hierarchical time series model to estimate and project the provincial SRB, with a focus on modelling the potential SRB imbalance.

**Results:**

In 2016, the highest SRB is estimated in Province 5 (*Lumbini Pradesh*) at 1.102, corresponding to 110.2 male births per 100 female births, with a 95% credible interval (1.044, 1.127) and the lowest SRB is in Province 2 at 1.053 (1.035, 1.109). The SRB imbalance probabilities in all provinces are generally low and vary from 16% in Province 2 to 81% in Province 5 (*Lumbini Pradesh*). SRB imbalances are estimated to have begun at the earliest in 2001 in Province 5 (*Lumbini Pradesh*) with a 95% credible interval (1992, 2022) and the latest in 2017 (1998, 2040) in Province 2. We project SRB in all provinces to begin converging back to the national baseline in the mid-2030s. By 2050, the SRBs in all provinces are projected to be around the SRB baseline level.

**Conclusions:**

Our findings imply that the majority of provinces in Nepal have low risks of SRB imbalance for the period 1980–2016. However, we identify a few provinces with higher probabilities of having SRB inflation. The projected SRB is an important illustration of potential future prenatal sex discrimination and shows the need to monitor SRB in provinces with higher possibilities of SRB imbalance.

**Supplementary Information:**

The online version contains supplementary material available at 10.1186/s12889-022-12693-0.

## Background

Under natural circumstances, the sex ratio at birth (SRB), the ratio of male to female live births, fluctuates within a narrow range, from 1.03 (i.e., 103 male births per 100 female births) to 1.07, with minor disparities due to maternal weight, [1] paternal age and birth order, [2] ethnicity groups such as Indians, [3] Africans, [4–6] Orientals, [7] Causations, [8] environmental conditions during pregnancy, [9, 10] country-specific circumstances in developed countries such as the United States [11, 12], Canada and Denmark [13]. However, since the 1970s, skewed and masculinized SRB has been reported in several countries worldwide, but mainly concentrated in Asia [13–17] and Eastern Europe, such as Hong Kong, [18] China, [17, 19–24] India, [16, 19–21, 25] Taiwan, [26, 27] Republic of Korea, [21, 28, 29] Caucasus, [30, 31], and Vietnam [32–35]. SRB imbalance is a direct result of sex-selective abortion; in short, “sex selection”. The action of sex selection in a certain population is largely driven by three factors [15, 16]. First, the population has a strong and sustained preference for sons. Second, technologies offering prenatal sex determination and abortion are accessible and affordable. Third, the number of children a woman gives birth to is decreasing as a result of fertility decline [36]. This fertility transition leads to a “fertility squeeze”, in which sex selection is used to obtain a desired small family and ensures male offspring.

In Nepal, the three main factors leading to sex selection have been documented. First, the predominant religion in Nepal is Hinduism, and being a patriarchal society, it attributes higher value to sons than daughters. This son preference in Nepal is reflected in various aspects: a stronger desire to have sons rather than daughters [37]; among women who decide to stop or have ceased childbearing, a higher proportion of the last born children are males rather than females [37, 38]; a skewed sex ratio for a higher order of births given previous female births [39–41]; and better access to education for boys compared to their sisters [42]. Previous studies have also found higher-than-expected mortality rates among girls under 5 years old in Nepal, which suggests the disadvantageous treatment of girls compared with boys at the postnatal stage [43]. Second, the technology of sex-selective abortion is accessible to the Nepalis. Even though prenatal sex determination and abortion based on sex selection are prohibited and may incur severe penalties in Nepal under the current abortion law passed in 2002, [44, 45] studies show an emergence of sex-selective abortions being conducted in some areas of Nepal [46]. Even before abortion was made legal, Nepali women practiced sex-selective abortion across the border in India [47]. Nepal and India share an open border, allowing the free movement of people. The majority of Nepal’s population live either near the border with India or within easy access via roads. Third, fertility has been declining in Nepal, with the total fertility rate (TFR), or children per woman, falling from 6.0 in 1950 to 1.9 in 2019 according to the UN [48]. The fertility rate in Nepal is expected to decline further since female educational attainment is increasing (e.g. among 20- to 24-year-olds, the proportion of females with at least lower secondary school educational attainment increased from 41% in 2005 to 57% in 2015), [49] and there is a negative relationship between education and fertility. In 2016, the TFR was 1.8 among women who had completed the tenth grade and 3.3 among those without education [50]. The relationship between education attainment and SRB imbalance is more complicated. It is highly likely that the higher educated women, who are also affluent and desire fewer children, were the first adopters of the idea of sex-selective abortions triggering the SRB inflation. However, the higher educated, increasing number of more emancipated women would later start correcting the practice of sex-selection abortions pioneered by their predecessors. Less-educated women will follow the practice as their fertility declines and trigger SRB inflation. Moreover, the return to the normal SRB level will likely be much slower than among the more educated women.

It is crucial to monitor and project the SRB at the subnational level for Nepal, since the national level may mask SRB imbalances in subpopulations. No prior study has found evidence of an ongoing sex ratio transition in Nepal on the national level. On the subnational level, however, studies have suggested that sex-selective abortion may exist in certain areas or a subgroup of the population in Nepal [39–41, 51]. Particularly, the study [40] identified 11 Nepali districts with skewed SRB and found that sex-selective abortion concentrated in Kathmandu Valley and Lumbini Province, based on the 2011 census. On the subnational level, Nepal has high levels of heterogeneity in terms of its demographics, socioeconomics, and culture (i.e. religion, caste, and ethnicity). The majority of Nepalis are Hindus (81%), followed by Buddhists (9%), Muslims (4.4%), Kirats (3%), Christians (1.4%), and others [52]. Moreover, Hindus are further divided by caste, namely, Brahman, Chhetri, Vaisya, Shudra, and Dalit. Furthermore, more than one religion is practiced within each ethnic group, and the distribution varies. Altogether, there are 125 caste and ethnic groups, with 123 spoken languages; 19 languages are spoken by 96% of the population [52]. In Nepal, some caste and ethnic groups (i.e. Dalit and many indigenous communities called, in Nepali, *Adibasi* and *Janajati*) are defined by the constitution as marginalized populations, with lower socioeconomic status and a history of subjection to oppression compared to higher castes (e.g. Brahman/Chhetri among the Khas ethnicity in the Hills and Madhesi ethnicity in Terai) and ethnicity [53]. As a result, not only do fertility levels differ between geographical locations and urban–rural areas, [50] the son preference intensity also differs across castes, ethnic groups, religions, and cultures [54]. The disadvantaged non-Dalit Terai castes have the highest level of son preference among men, whereas the least son preference is reported among the Brahman/Chhetri [54]. The association between eco-developmental regions and provincial SRB is explained mainly by population heterogeneity, socioeconomic and cultural, such as educational attainment, ethnicity, and religion. While the predominant religion in Nepal was Hinduism (81% in 2011) followed by Buddhists (9%) and Islam (4.4%), the proportion varies by the ecological regions, namely, Mountain (69% Hindu, 24% Buddhists, and less than 1% Muslim), Hill (80, 13, and < 1%), and Terai (85, 3, and 9%). Since the son preference is usually very high among the Hindus, [55] one can expect higher son preference in Terai and least in the Mountains. Also, ethnicity is associated mainly with religion, and certain ethnic groups (*Janajati* or indigenous, despite being Hindus), have lower son preference.

Nepal established a new federal system with seven provinces (called *Pradesh*) and 753 municipalities in September 2015, with the first elections for the federal and provincial parliaments held in 2017. The seven provinces^1^ each act as prime administrative units for policymaking. Most provinces include multiple ecological zones: (i) Mountain in the northern Himalayan belt, (ii) Hill in the central region, and (iii) Terai in the southern plain. Exceptions are Province 2, entirely in Terai; Province 5 (*Lumbini Pradesh*), which lacks Mountain; and Province 6 (*Karnali Pradesh*), which lacks Terai. Table 1 presents Nepal’s provincial profiles, including population distribution by ecological zones, by caste/ethnicity and religion, GDP per capita, and education status. It is crucial to estimate and project the SRB imbalance in Nepal by province to directly assist the monitoring of population indicators and program planning, in order to address the huge heterogeneities across provinces in demography, education, ecology, and culture as shown in Table 1.

**Table 1:** Demographic, socioeconomic and cultural characteristics by Nepal Province. The population distribution by ecology zone, education attainment, caste/ethnicity and religion are from Census 2011. Young adult refers to 20–39 years old. GDP during 2018–2019 is from [56], using an exchange rate of 1 USD = 121.6 NPR

Nepal Province	Population distribution by ecological zones				Young adults with at least lower secondary	GDP per capita 2018–2019	Population distribution by caste/ethnicity and religion						
(province name)	Total (in millions)	Terai	Hill	Mountain		(in USD)	Adivasi-Terai	Janajati-Hill	Khas Hindu (non- Dalits)	Dalit Hindu	Madhesi Hindu (non- Dalits)	Muslim	others
Province 1	4.53	56%	35%	9%	43%	942	9%	42%	28%	9%	8%	3%	1%
Province 2	5.40	100%	0%	0%	19%	657	5%	5%	5%	15%	55%	13%	2%
Province 3 (Bagmati)	5.53	10%	80%	9%	49%	1744	1%	62%	31%	5%	1%	0%	0%
Province 4 (Gandaki)	2.41	13%	86%	1%	45%	1018	1%	50%	32%	16%	0%	1%	0%
Province 5 (Lumbini)	4.50	72%	28%	0%	31%	845	14%	19%	27%	15%	17%	7%	1%
Province 6 (Karnali)	1.56	0%	75%	25%	20%	768	0%	17%	64%	18%	0%	0%	0%
Province 7 (Sudurpashchim)	2.55	48%	34%	18%	22%	761	11%	3%	64%	19%	2%	0%	0%

This study aimed to estimate the levels and trends in SRB for the seven Nepal provinces between 1980 and 2016 and provide probabilistic SRB projections^2^ up to 2050. The paper is organized as follows: we first explain the data sources and the Bayesian statistical model for SRB estimation and projection. We then present the modeled results for SRB by province over time. In the discussion, we summarize the main contributions, limitations due to data availability and model assumptions, and suggestions for future research.

### Data and methods

The data and method details, including the preprocessing of data, motivations of model choices, model specifications, priors, statistical computation, and validation approaches and their results are available in the Additional file 1. We summarize the main steps in the rest of the section.

### Data

We compiled an SRB database for the seven Nepal provinces. The database consists of 92 province-level SRB observations with reference years ranging from 1976 to 2016. The total number of births involved in constructing the SRB database is 152,355. Table 2 summarizes the database by data source. The database is available as the Additional file 2.

**Table 2:** SRB database by source. The size of birth samples reported in the table refers to the unweighted total number of births within the 25 years prior to when the surveys were conducted

Data source	# SRB observations	Size of birth samples
NDHS 2001	22	27,958
NDHS 2006	21	25,952
NDHS 2011	21	26,012
NDHS 2016	21	25,592
Census 2011	7	46,841
total	91	152,355

We obtained the SRB observations from survey and census microdata.^3^ For the survey data, we included the 2001, 2006, 2011, and 2016 Nepal Demographic and Health Surveys (NDHSs). NDHSs are retrospective surveys which obtain full birth histories (all births that a woman has given in her life so far) from women aged 15–49. We did not include the 1996 NDHS or the 1976 World Fertility Survey because the microdata files do not contain the respondents’ district information; we were unable to extract SRB by province from these two surveys. To obtain NDHS observations with associated uncertainty at a controlled level, we merged observations with initially one-year period into a multi-year period based on the coefficient of variation for SRB (Additional file 1 section 1.2) [57]. We then computed the sampling errors for the observations using a jackknife method for the four NDHSs to reflect the uncertainty resulting from the multi-stage stratifying sample design (Additional file 1 section 1.2) [58–60]. We excluded NDHS observations that are more than 25 years prior to the survey year to minimize recall bias for the sex composition of older females’ full birth history. We included the 2011 Census in the database where the birth file included the sex of the last birth within 12 months prior to the census. The microdata sample from the census consists of around 15% (4 million individuals) of the population, with a coverage range of 11–99% of the population from each district. When aggregating the district-level birth data to the provincial level, we applied weights to the births data by district according to the proportion of the sampled population by district to adjust for over- and under-sampling. For both the NDHS and the census data, to obtain provincial SRB observations, we aggregated the sex-specific births from 75 districts according to the province they belonged to (Additional file 1 section 1.1), except for the 2016 NDHS, since it already contained province information in the microdata file. We excluded the 2001 Census from our analysis due to its implausible data quality at the district level.

We compiled the TFR (as model input, which will be explained in Methods) by province from the 2001, 2006, 2011, and 2016 NDHSs (Additional file 1 section 1.3) using R-package DHS.rates [61, 62]. For the 2001 NDHS, because the survey respondents were “ever-married females” and those who were never married at the time of the survey were not included, we weighted each female respondent using an “all-women factor” so that the resulting microdata represented responses from all women instead of only females who were ever married [63]. The TFR for the period beyond 2016 was based on a medium-fertility scenario of the population projections for the 753 municipalities [64]. We then aggregated the municipality births and the women’s years of exposure to the reproducible periods by province to generate the provincial TFR.

## Methods

We used a Bayesian hierarchical time series model to estimate and project the SRB by province, with a focus on modeling the potential SRB imbalance due to sex selection up to 2050. The model is largely based on the one as described in a prior study [65]. However, we made a few modifications to the model in this study to produce improved fit for the provincial SRB observations in Nepal. We briefly summarize the model here. The full model details are in Additional file 1 section 2. The sensitivity analyses are in Additional file 1 section 5, where we show that the model results are not sensitive to the model settings.

We define Θ_*p*, *t*_ as the SRB for a Nepal Province *p* in year *t*. We model Θ_*p*, *t*_ as:

1 $${\Theta}_{p,t}=b{\Phi}_{p,t}+{\delta}_p{\alpha}_{p,t}.$$

The first part of the summation *b*Φ_*p*, *t*_ models the year-by-year fluctuations of the underlying reference level of SRB. We fix *b* = 1.049, taken from a previous study, [37] is the SRB baseline level for the entire country, assumed to be known and constant across provinces over time; Φ_*p*, *t*_ captures the natural fluctuations around the national SRB baseline *b* and is modeled as a first-order auto-regressive model, i.e. AR (1), on the log scale.

**Fig. 1:**
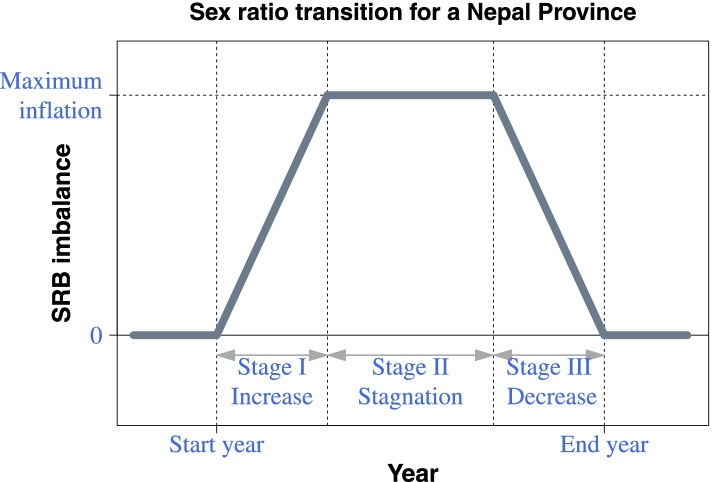
Illustration of sex ratio transition model for a Nepal Province

The second part of the summation *δ*_*p*_*α*_*p*, *t*_ models the provincial-specific sex ratio transition process with transition probability that varies across provinces. *δ*_*p*_ is the province-specific binary parameter that detects the absence or presence of SRB inflation: *δ*_*p*_ = 0 refers to no SRB inflation (relative to the national baseline *b*) for province *p*; and *δ*_*p*_ = 1 indicates the existence of imbalanced SRB. We assumed that *δ*_*p*_ follows a Bernoulli distribution with province-specific means. The SRB inflation probability by province was computed as the number of posterior samples with *δ*_*p*_ = 1 divided by the total number of posterior samples. *α*_*p*, *t*_ models the SRB inflation process over time for province *p* in year *t*; it was assumed to be non-negative and to capture the upward skewed SRB due to sex-selective abortion [16]. Assuming a trapezoid function according to the study, [65] we modeled the period lengths of three stages of a sex ratio transition (i.e. increase, stagnation, and convergence back to the national SRB baseline) and the maximum level of SRB inflation. Fig. 1 illustrates the trapezoid function for *α*_*p*, *t*_ that we use to model the three stages of the SRB inflation process. All the shape parameters related to the trapezoid function (i.e. start year, period lengths of three stages of SRB transition, and maximum level of imbalance) were province-specific and followed hierarchical distributions. We assigned informative priors to the mean and variance of these hierarchical distributions, which were based on the national-level sex ratio transition experience in Nepal [65]. The province-specific start year of the SRB inflation incorporated the fertility squeeze effect by using the TFR. The start year parameter followed a Student-*t* distribution with three degrees of freedom. The choice of a heavy-tail distribution is to enable the model to capture the start years of an SRB imbalance with potential outlying TFR among the provinces. The mean of the start year distribution followed the relationship between national SRB imbalance and fertility decline. Please refer to the Additional file 1 section 2 for more details on specifications of each model element.

The model took into account varying uncertainties associated with the observations. For the observed SRB on the log scale, log(*r*_*p*, *t*_), at province *p* in year *t*, we assumed that it follows a normal distribution:

2 $$\log \left({r}_{p,t}\right)\mid {\Theta}_{p,t}\sim \mathcal{N}\left(\log \left({\Theta}_{p,t}\right),{\sigma}_{p,t}^2\right),$$

where *σ*_*p*, *t*_ is the sampling error for log(*r*_*p*, *t*_), which reflects the uncertainty in the observations due to survey sampling design. Each *σ*_*p*, *t*_ represents the data quality of the corresponding observation. The smaller the value of *σ*_*p*, *t*_ the higher quality of the data point. Consequently, the estimated Θ_*p*, *t*_ is pooled more toward observations with smaller *σ*_*p*, *t*_ than bigger *σ*_*p*, *t*_. It was pre-computed using a jackknife method as explained in the Data section (and in details in Additional file 1 section 1.2).

The model performance was assessed using an out-of-sample validation exercise and a simulation analysis (Additional file 1 section 3). Due to the retrospective nature of the SRB data and the occurrence of data in series, we left out 20% of the observations. Instead of randomly selecting the left-out observations, we select those data that were collected since 2016 in the out-of-sample validation exercise. This approach has been used in other studies to validate the model’s predictive power for demographic indicators based on retrospective information [43, 66–68]. The validation and simulation results suggested that our model was reasonably well-calibrated, with generally conservative credible intervals (Additional file 1 section 4). The sensitivity analyses show that the model results are not sensitive to model assumptions (Additional file 1 section 5).

## Results

The SRB estimates and projections from 1980 to 2050, including uncertainty for the seven Nepali provinces, are presented in the Additional file 3 and Additional file 4.

### Levels and trends in SRB between 1980 and 2016 by province

**Fig. 2:**
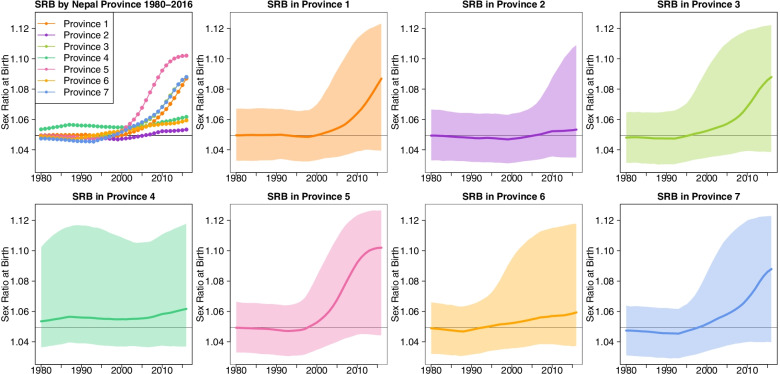
SRB estimates for seven provinces of Nepal during 1980–2016. The median estimates are shown in the top left panel across all provinces. In all the rest panels, median estimates are in curves and 95% credible bounds are in shades. Horizontal line indicates the national SRB baseline value at 1.049 [37]

**Fig. 3:**
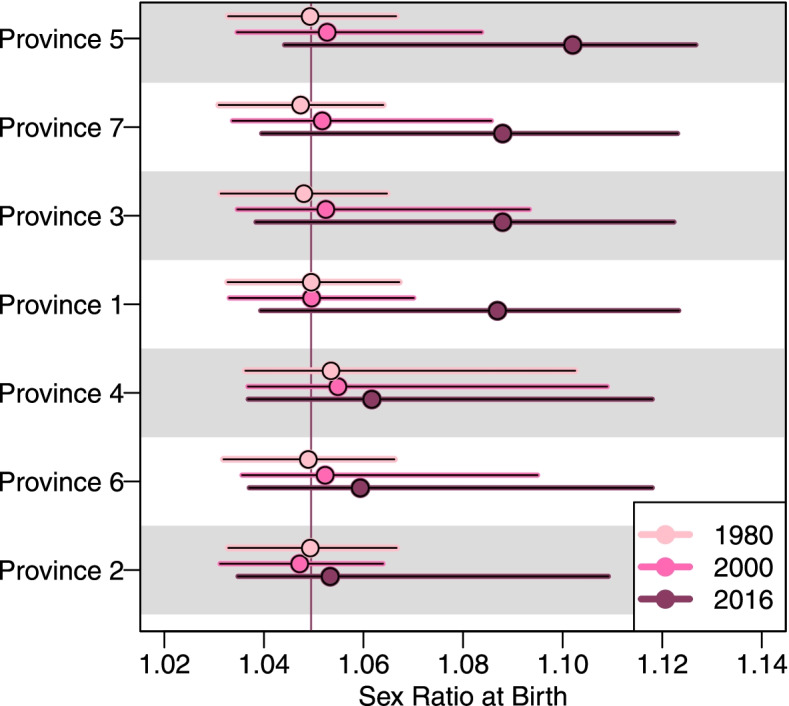
SRB by Nepal province in 1980, 2000 and 2016. Dots refer to the median estimates. Line segments are the 95% credible intervals. Vertical line indicates the national SRB baseline value at 1.049 [37]. Provinces are in descending order of the median estimates of SRB in 2016

Fig. 2 provides an overview of the estimated levels and trends in SRB for the seven provinces from 1980 to 2016. The modeled estimates imply more disparities in the provincial SRB after 2000 than before 2000. In 2016, the estimated SRB is the highest in Province 5 (*Lumbini Pradesh*), at 1.102 with a 95% Bayesian credible interval (1.044, 1.127), and it is the lowest in Province 2, at 1.053 (1.035, 1.109) as shown in Fig. 3. None of the provinces has an SRB that is statistically significantly different from the national SRB baseline level of 1.049 (i.e. the baseline SRB for the whole of Nepal was taken from [37]). Before 2000, the SRB remains around the national SRB baseline with minor natural fluctuations in all provinces. In 1980, the SRB ranges from 1.047 (1.031, 1.064) in Province 7 (*Sudurpaschhim Pradesh*) to 1.053 (1.036, 1.103) in Province 4 (*Gandaki Pradesh*).

### SRB imbalances by province

Table 3 summarizes the modeled results of the SRB imbalances for the seven provinces. The start years for the SRB imbalances are estimated to range from 2001 in Province 5 (*Lumbini Pradesh*) with a 95% credible interval (1992, 2022) to 2017 (1998, 2040) in Province 2. The TFR at the start of the SRB inflation ranges from 2.6 (i.e. the average number of children born per woman) in Province 2 and 2.7 in Province 6 (*Karnali Pradesh*) to as high as 3.9 in Province 7 (*Sudurpashchim Pradesh*) and 4.4 in Province 5 (*Lumbini Pradesh*).

**Table 3:** SRB imbalance by Nepal province. The median estimates of SRB inflation start year are in front of brackets. The 95% credible intervals for the start year are in brackets. The TFR values in the median estimates of start year are reported. The province names are in brackets if available

Nepal Province (province name)	SRB inflation start year	TFR in start year	SRB inflation probability
Province 1	2006 (1994, 2028)	3.1	62%
Province 2	2017 (1998, 2040)	2.6	16%
Province 3 (Bagmati)	2004 (1989, 2026)	3.5	63%
Province 4 (Gandaki)	2006 (1976, 2029)	3.0	55%
Province 5 (Lumbini)	2001 (1992, 2022)	4.4	81%
Province 6 (Karnali)	2013 (1989, 2035)	2.7	35%
Province 7 (Sudurpashchim)	2005 (1991, 2028)	3.9	62%

The probabilities of having an SRB imbalance varies for all provinces, but it is generally low, ranging from 16 and 35% in Province 2 and Province 6 (*Karnali Pradesh*), respectively, to 81% in Province 5 (*Lumbini Pradesh*). The average inflation probability for the seven provinces is 53%. These findings are in line with previous studies that found no SRB imbalance on the national level.

### Probabilistic projections of SRB between 2016 and 2050 by province

**Fig. 4:**
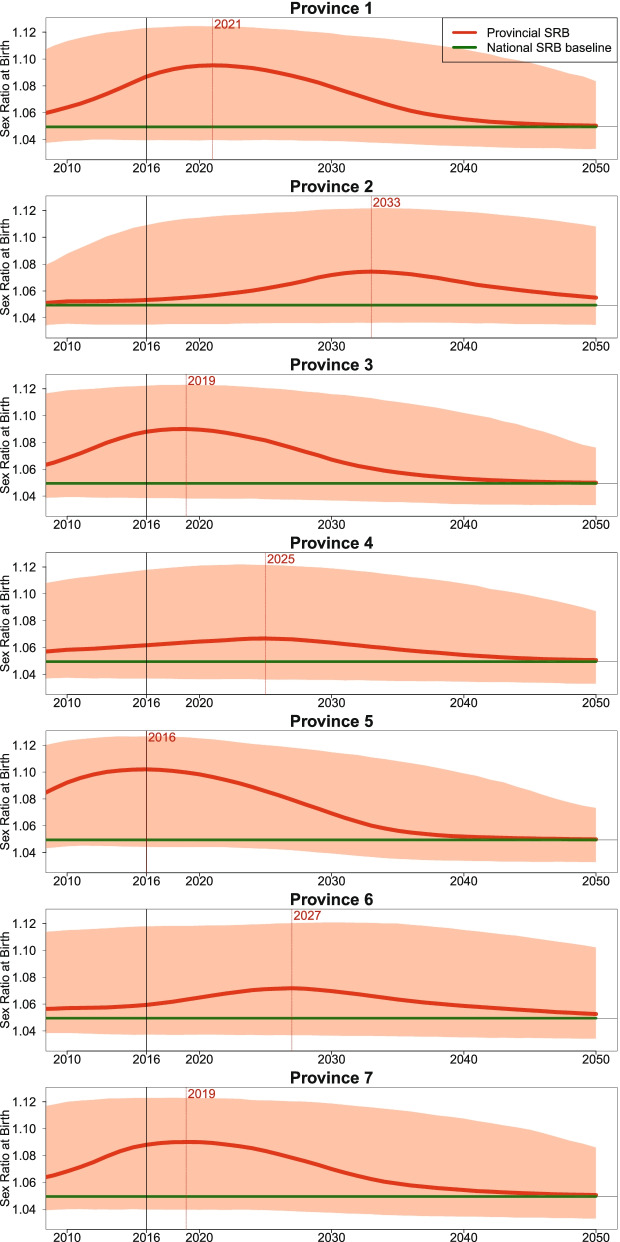
SRB projections for 7 Nepal provinces during 2016–2050. Median projections of provincial SRB (red curve), 95% credible interval (red shade), national SRB baseline value at 1.049 (green horizontal line) [37]. The start years of SRB inflation are shown in black solid vertical lines. The year in which the median projection reaches the maximum in each province is shown in red dashed vertical line with the year indicated in red on top

**Fig. 5:**
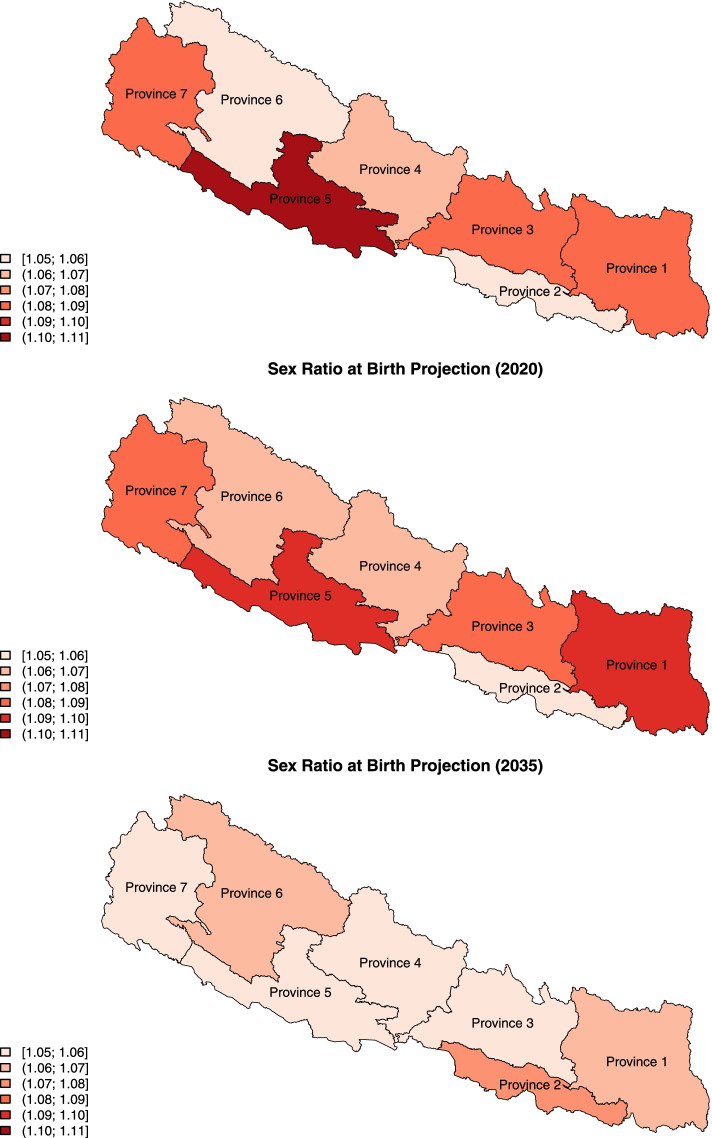
Projected SRB by Nepal province. ﻿The median SRB projections are shown by Nepal province in 2016 (top), 2020 (middle) and 2035 (bottom) [69]

During the period 2016–2050, the levels, trends, and imbalances of SRB in Nepal are projected to differ across the provinces, given the model assumptions of province-specific probabilities having SRB inflation (see Fig. 4 and Fig. 5). At the beginning of the projection period since 2016, the SRB imbalances are projected to start declining to the national SRB baseline in Province 5 (*Lumbini Pradesh*), 3, and 7. The sex ratio transitions in Province 1 and 4 are in the midst of climbing to the maximum levels of SRB imbalance, and the SRB inflation has just started in Province 2 and 6. The year in which the projected SRB reaches its maximum ranges from 2016 in Province 5 (*Lumbini Pradesh*), with an SRB of 1.102 (1.044, 1.127), to 2033 in Province 2, with an SRB of 1.074 (1.036, 1.122). Around the mid-2030s, the SRB in all the provinces is projected to start converging back to the SRB national baseline. By 2050, the SRB in all the provinces is projected to be around the baseline level.

## Discussion

To the best of our knowledge, this is the first study to estimate and project SRB by Nepal province from 1980 to 2050. The database of province-level SRB in Nepal used for this study is by far the most extensive to date; it includes four NDHSs and one census, covering a total of 152,355 birth records. We adopted a Bayesian hierarchical time series model from a prior study (with modifications) to capture natural sex ratio fluctuations around the national baseline for each province, model the sex ratio transition with a province-specific probability of having SRB inflation, and account for varying uncertainties associated with the observations [65]. The model captured regularities in sex ratio transition patterns across the provinces and incorporated the TFR to estimate the start year of the sex ratio transition process to capture the fertility squeeze effect. With the Bayesian hierarchical setup, we were able to use information about the national-level sex ratio transition from a previous study to assist in estimating provincial-level SRB imbalances [65]. Based on the model assumption of the province-specific probability of having an SRB imbalance, we projected the sex ratio transitions and resulting imbalanced SRB across the provinces. Consequently, the SRB projections for the seven provinces are model-based and data-driven.

The model results imply that there have been more disparities in SRB across the provinces since 2000, even though the provincial SRBs are not estimated or projected to be statistically significantly different from the national baseline. We estimate that the probability of having an existing SRB imbalance varies greatly among the seven provinces, from 16% in Province 2 to 81% in Province 5 (*Lumbini Pradesh*). In Province 5 (*Lumbini Pradesh*), SRB inflation is estimated to start when the TFR declines to 4.4, which is the highest TFR at the start of the sex ratio transition among all the provinces. These findings make Province 5 (*Lumbini Pradesh*) unique in the context of sex ratio imbalance at birth. A possible explanation for this is its geographical location; the province borders India, a country with a strong son preference and an ongoing SRB imbalance. In addition, Province 5 (*Lumbini Pradesh*) contains mostly Terai (72% of the population, as of 2011), and the son preference is relatively higher among Terai ethnic populations compared to the Hills [54]. According to a previous study, the TFR was around 5.2 when the SRB in India started to inflate [37]. As for Province 2, it has the lowest probability of experiencing an ongoing SRB imbalance according to our results. Although it is geographically and ethnically close to Bihar (100% of the population reside in Terai, and 83% of the population are Madhesi, Muslim, or Dalits), an Indian state with a strong son preference and imbalanced SRB, [25] Province 2 is one of the least developed provinces in Nepal, given it has the least per capita income (see Table 1). It could be that abortion technology is not as affordable or accessible in Province 2 as it is in richer provinces. This speculation is in line with studies that have shown that although the established fee for an abortion service in the public sector is around US$8–14, [70, 71] hidden costs for medications, materials (such as surgical gloves), and equipment (such as syringes), may be beyond the means of poor or marginalized women [72].

Our SRB estimates and probabilistic projections are based on several modeled assumptions and are, therefore, subject to limitations. First, in the sex ratio transition model, we only incorporated the fertility squeeze effect. In particular, we assume that the fertility transition in Nepal affects the start year of the sex ratio transition, not on other elements of the SRB inflation process (e.g. maximum level of the inflation, period lengths of the increase, stagnation and decrease of the inflation process). We did not incorporate any additional factors that may affect SRB imbalance. Several studies have considered the son preference effect on SRB inflation, [73–75] but they are either simulation studies that do not estimate and project SRB or the proxy indicator for son preference intensity is based on much bigger population sizes. Conditional SRB in Nepal has also been reported to associate with sex-selective abortion [39, 40]. We do not incorporate this factor in the Bayesian model due to the lack of reliable projections on the conditional SRB by Nepal province up to 2050. When looking at these indicators by Nepal province, the values are not informative enough since the sample sizes are too small. Second, instead of modeling global parameters related to the natural fluctuation of SRB in the time series model and global parameters (i.e. not province-specific parameters) related to the sex ratio transition process, we borrowed such information from prior studies [37, 65]. When we attempted to model all these global parameters, the resulting SRB had too much uncertainty to provide any meaningful trends. Hence, we focused the model on the provincial SRB imbalance and assumed the national sex ratio transition experience followed from previous studies. Third, we model that the SRB imbalance is nonnegative because we assume that the SRB imbalance mainly happens in the context of son preference instead of girl preference. Given that Nepal is a country with strong son preference, we do not consider the possibility of SRB deflation. Forth, the uncertainty bounds for the projections in some of the province-years are wide, even after we used informative priors for sex-ratio-transition-related parameters. This was mainly due to the small birth sample sizes for each province from data sources, which resulted in relatively large uncertainties for the observations. Lastly, when interpreting the projected SRB, it is important to bear in mind that the projection was made under the assumption that the SRB will inflate in the future and follow the national experience of sex ratio transition with a probability. On average, the provincial-level sex ratio transition is covered with data for 15.5% of the total transition period, corresponding to 5.5 out of 35.1 years across seven provinces. Hence, the national transition experience with a span of 36 years has significant influence on projecting the provincial SRB inflation, especially for the last two stages of the sex ratio transition, resulting all provincial transition ends by 2050. Applying other model assumptions may result in slightly different trajectories for projected SRB as we have shown in our sensitivity analyses (Additional file 1 section 5). However, none of these differences are statistically significantly different from zero, for both the estimation and projection periods.

We assume three stages of sex ratio transition in our model with increase, stagnation, and decrease back to normal. The non-linear pattern is admittedly more complicated than the assumptions used in other studies: remain constant after a certain year [76] or increase linearly [77]. However, the three stages of sex ratio transition are supported by data. They have been fully or partially observed in countries/areas such as Albania, Armenia, Azerbaijan, China, Georgia, Hong Kong, India, Republic of Korea, Montenegro, Taiwan, and Tunisia [78]. Previous SRB projections for Nepal assume simple monotonic convergence [48, 64, 77]. The national population projection by the Central Bureau of Statistics of Nepal [79] for the period 2011–2031 assumed that the SRB would be 1.07 in 2011 (based on DHS estimates) that will increase to 1.11 by 2031 (converging to the SRB in India in 2011) [79]. UN (2010–2100) assumes 1.05 for 2010–2015, increasing to 1.07 by 2015–2020 and stabilizing [48]. At the sub-national level, the SRB projection done by KC et al. 2016 assumes the district level SRB indirectly estimated from Census 2011 to converge to 1.05 by 2051 [64]. In the cohort-component population projection method, SRB projection is essential in splitting the projected births into males and females. The implication of different SRB projections primarily unfolds when the daughters enter their reproductive ages such that larger deviance in SRB could result in significantly different total births. Based on our model, we expect that the SRBs in different regions do not change monotonically. Rather, we assume a non-linear three-stage transition process for the SRB (increase, stagnation, and decrease), based on patterns observed in other countries with existing SRB imbalance [65, 78]. Population projection using our SRB projection would be analytically more advantageous and realistic than the ones that assumes monotonic convergence. Hence, the three-stage SRB inflation is necessary and suitable to apply to Nepal.

Contrary to earlier centralized policymaking in Nepal, the new federal system has devolved the health sector to provincial- and municipality-level governments. While the system is under development, we can expect greater differences in policies, implementation, and responses from individuals. Our results show that SRB levels and trends and the probabilities of SRB inflation vary greatly across the provinces. The projected provincial SRB inflation is meant to illustrate a hypothetical future if no changes in policy or current intervention, in addition to model assumptions. Our projection results provide a helpful guideline for policymakers on anticipating the level of female birth deficits based on the current situation. Therefore, it is essential to strengthen existing policies and devise new ones, considering the multiple layers of governance in the new federal system.

Future studies could make use of the projected SRB and calculate the number of missing female births to quantify the effect of the imbalanced SRB by Nepali province when the number of births by province are made available [80]. Policymakers can devise interventions that successfully reduce the SRB inflation, such as strict laws (forbid the abuse of the technology for sex selection), changing gender-related social norms by empowering girl children, banning compulsory dowry, and so on. Nepal has already in place many of these policies. For example, in 2002, sex-selective abortions were criminalized (Act: Chapter 10, Number 28C) [81]. More recently, the Province 2 government has implemented ‘*Beti Bachau – Beti Padhau*’ (Save daughter - educate daughter) scheme by starting insurance for every newly born girl, who will receive an amount after reaching a certain age [82]. Our analysis identified areas where the SRB inflation could occur in the future, and this information can be used in policy planning to strengthen the implementation of the policies and optimize resource usage to prevent future female birth deficits. As we estimated the probability of SRB inflation for each province, in-depth studies of provinces and municipalities, conditioning on the availability of reasonably good-quality data, are required to monitor and confirm whether a sex ratio imbalance at birth has occurred or is ongoing. Future research, including field studies in collaboration with the government(s) and non-governmental organizations (NGOs)/ international non-governmental organizations (INGOs), would be useful for collecting high-quality data from subpopulations to better monitor prenatal sex discrimination in Nepal.

## Conclusions

Our Bayesian hierarchical model results suggest that on average, the risk of having SRB imbalance across Nepal provinces is low at 53% from 1980 to 2016. Province 5 (*Lumbini Pradesh*) is estimated to have the highest probability of undergoing SRB inflation at 81% during the estimation period. Based on observed SRB data across Nepal provinces prior 2016 as well as the model assumption of three-stage of sex ratio transition, the Bayesian hierarchical model projects potential imbalanced SRB till 2050. The maximum SRB imbalance within each province is projected to occur the soonest in Province 5 (*Lumbini Pradesh*) in 2016 and the latest in Province 2 in 2033. The SRB probabilistic projections illustrate that potential future prenatal sex discrimination may differ across Nepal provinces. The projections also provide useful guidelines for policy planning and risk assessment in provinces with higher possibilities of SRB imbalance.

## Supplementary Information

**Additional file 1.** Technical appendix which includes details on data preprocessing, model specifications and motivations, statistical computing, validation approaches and results, and sensitivity analyses. DOI: https://doi.org/10.6084/m9.figshare.19077155 [83].

**Additional file 2.** Nepal provincial SRB database. DOI:https://doi.org/10.6084/m9.figshare.18765881 [85].

**Additional file 3.** SRB estimates by Nepal province from 1980 to 2016. DOI: https://doi.org/10.6084/m9.figshare.18771695 [86].

**Additional file 4.** SRB projections by Nepal province from 2017 to 2050. DOI:https://doi.org/10.6084/m9.figshare.18772877 [87].

## Data Availability

Additional file 1 (technical appendix) is available from the figshare repository, DOI: 10.6084/m9.figshare.19077155 [83]. The DHS microdata files are available from The DHS Program website https://dhsprogram.com/ [84]. The Nepal provincial SRB database is available as Additional file 2, DOI:10.6084/m9.figshare.18765881 [85]. The SRB estimates by Nepal province from 1980 to 2016 is available as Additional file 3, DOI: 10.6084/m9.figshare.18771695 [86]. The SRB projections by Nepal province from 2017 to 2050 is available as Additional file 4, DOI:10.6084/m9.figshare.18772877 [87].

## Abbreviations

AR (1): First-order auto-regressive model
INGO: International non-governmental organization
NDHS: Nepal Demographic and Health Survey
NGO: Non-governmental organization
SRB: Sex ratio at birth
TFR: Total fertility rate

## Footnotes

1 To date, five province-level parliaments have decided the names of their provinces. In this study, we use Province 1 to 7 to label the seven provinces and provide the province names if available.

2 The SRB observations are available up to 2016. Hence, results beyond 2016 are considered projections in this study.

3 In this study, we use the term “microdata” to refer to individual-level data collected in surveys and censuses.
