# Supplementary material for: Estimation and probabilistic projection of levels and trends in the sex ratio at birth in seven provinces of Nepal from 1980 to 2050: a Bayesian modeling approach
Source: BMC Public Health. 2022 Feb 19;22:358. doi: 10.1186/s12889-022-12693-0 (PMC8858562; doi:10.1186/s12889-022-12693-0)
Supplement: Supplementary file 1 — Additional file 1. Technical appendix which includes details on data preprocessing, model specifications and motivations, statistical computing, validation approaches and results, and sensitivity analyses. DOI: https://doi.org/10.6084/m9.figshare.19077155 [83]. [file 12889_2022_12693_MOESM1_ESM.pdf]

Technical Appendix for
Estimation and probabilistic projection of levels and trends in the sex ratio at birth in seven provinces of Nepal from 1980 to 2050: a Bayesian modeling approach

Fengqing Chao*[1], Samir K.C.†[2,3], and Hernando Ombao[1]

[1]Biostatistics Research Group, Statistics Program, Computer, Electrical and Mathematical Sciences and Engineering Division, King Abdullah University of Science and Technology (KAUST), Thuwal, Saudi Arabia

[2]Asian Demographic Research Institute, Shanghai University, Shanghai, China

[3]Wittgenstein Centre for Demography and Global Human Capital (IIASA, VID/OeAW, UV), International Institute for Applied Systems Analysis, Laxenburg, Austria

January 27, 2022

*Corresponding author (FC); Email: fengqing.chao@kaust.edu.sa
†Corresponding author (SKC); Email: kcsamir@gmail.com

Contents

1 Data Pre-processing **1**
1.1 Matching Nepal districts to provinces . . . 1
1.2 Sampling errors for DHS data . . . 1
1.3 TFR Data by Nepal Province . . . 2

2 Model **3**
2.1 Noations . . . 3
2.2 Model for Sex Ratio at Birth by Nepal Province . . . 3
2.3 Data quality model . . . 5
2.4 Priors . . . 6
2.5 Statistical Computing . . . 6

3 Model Validation **6**
3.1 Out-of-Sample Validation . . . 7
3.2 One-Province Simulation . . . 7

4 Validation and Simulation Results **7**

5 Sensitivity analyses **9**
5.1 Analysis for start year distribution . . . 9
5.2 Analysis for sex ratio transition variances . . . 12
5.3 Analysis for time series model . . . 16

6 Supplementary Figures **18**

List of Tables

1 Nepal districts and provinces matching . . . 1
2 Notation summary . . . 4
3 Validation and simulation results for left-out SRB observations . . . 8
4 Validation results for estimates based on training set . . . 8
5 Sensitivity analysis for t_{0p} . . . 9
6 Sensitivity analysis for $\Theta_{p,t}$ with different start year distributions . . . 11
7 Sensitivity analysis for $\Theta_{p,t}$, 1980–2050 . . . 12
8 Sensitivity analysis for $\Theta_{p,t}$, 1980–2016 . . . 13
9 Sensitivity analysis for $\Theta_{p,t}$, 2016–2050 . . . 13
10 Sensitivity analysis for ξ_p . . . 15
11 Sensitivity analysis for λ_{1p} . . . 15
12 Sensitivity analysis for λ_{2p} . . . 15
13 Sensitivity analysis for λ_{3p} . . . 15

List of Figures

1 SRB model results based on different start year distributions . . . 10
2 SRB model results based on different CV . . . 14
3 $\Phi_{p,t}$ model results based on AR(1) and AR(2) . . . 17

4 SRB estimates and projections by Nepal Province, 1980–2050 18

List of Abbreviations

NDHS	Nepal Demographic and Health Survey
MCMC	Markov chain Monte Carlo
SRB	Sex Ratio at Birth
TFR	Total Fertility Rate

1 Data Pre-processing

1.1 Matching Nepal districts to provinces

Nepal Demographic and Health Surveys (NDHS) 2001, 2006, 2011 and 2016 record the district information for each interviewed woman. We use the individual-level information on district to merge birth information to the 7 Nepal provinces. Table 1 summarizes the matching of the 75 Nepal districts to provinces. The Nawalparasi district is splitted into Province 4 and 5. We classify it in Province 5 because it is geographically closer. Rukum district is split into Province 5 and 6. In NDHS 2016, it is considered as in Province 6 and we follow this classification in our study.

Nepal Province	Province Name	[75] District
**Province 1**	–	[14] Bhojpur; Dhankuta; Ilam; Jhapa; Khotang; Morang; Okhaldhunga; Panchthar; Sankhuwasabha; Solukhumbu; Sunsari; Taplejjung; Terhathum; Udayapur
**Province 2**	–	[8] Bara; Dhanusa; Mahotari; Parsa; Rautahat; Saptari; Sarlahi; Siraha
**Province 3**	Bagmati	[13] Bhaktapur; Chitwan; Dhading; Dolakha; Kathmandu; Kavre; Lalitpur; Makwanpur; Nuwakot; Ramechhap; Rasuwa; Sindhuli; Sindhupalchowk
**Province 4**	Gandaki	[10] Baglung; Gorkha; Kaski; Lamjung; Manang; Mustang; Myagdi; Parbat; Syangja; Tanahu
**Province 5**	Lumbini	[11] Arghakhanchi; Banke; Bardia; Dang; Gulmi; Kapilbastu; Nawalparasi; Palpa; Pyuthan; Rolpa; Rupandehi
**Province 6**	Karnali	[10] Dailekh; Dolpa; Humla; Jajarkot; Jumla; Kalikot; Mugu; Rukum; Salyan; Surkhet
**Province 7**	Sudurpashchim	[9] Achham; Baitadi; Bajhang; Bajura; Dadeldhura; Darchula; Doti; Kailali; Kanchanpur

Table 1: **Nepal districts and provinces matching.** The red numbers at the beginning of each cell refers to the number of districts belong to each province. Currently, four province-level parliaments have decided the names of their provinces. In this study, we use Province 1 to 7 to label the seven provinces and provide the province names if available.

1.2 Sampling errors for DHS data

NDHS provide individual-level data with the full birth history for each women at reproductive age interviewed during the survey fieldwork period. We calculate the sampling error for log-transformed SRB for NDHS data series using the jackknife method [1, 2, 3]. For a certain NDHS, let U denote the total number of clusters. The u-th partial prediction of SRB is given by:

$$r_{-u} = \frac{\sum_{n=1}^{N} \mathbb{I}_n(x_n = \text{male}; d_n \neq u) w_n}{\sum_{n=1}^{N} \mathbb{I}_n(x_n = \text{female}; d_n \neq u) w_n}, \text{ for } u \in \{1, \ldots, U\},$$

where n indexes the live births in each state-survey-year, N is the total number of live births. x_n is the sex for the n-th live birth. d_n is the cluster number for the n-th live birth. w_n is the sampling weight for the n-th live birth. $\mathbb{I}_n(\cdot) = 1$ if the condition inside brackets is true and $\mathbb{I}_n(\cdot) = 0$ otherwise. The u-th pseudo-value

estimate of the SRB on log-scale is:

$$\log(r)^*_u = U\log(r') - (U-1)\log(r_{-u}), \text{ where}$$
$$r' = \frac{\sum_{n=1}^N \mathbb{I}_n(x_n = \text{male})w_n}{\sum_{n=1}^N \mathbb{I}_n(x_n = \text{female})w_n}.$$

The sampling variance is:

$$\sigma^2 = \frac{\sum_{u=1}^U (\log(r)^*_u - \overline{\log(r)^*_u})^2}{U(U-1)}, \text{ where}$$
$$\overline{\log(r)^*_u} = \frac{1}{U}\sum_{u=1}^U \log(r)^*_u.$$

For NDHS data, the annual log-transformed SRB observations are merged such that the coefficient of variation (CV) for log-transformed SRB is below 0.05 or the merged period reached 10 years [4]. For a certain NDHS data series, let $\{t_n, t_{n-1}, \cdots, t_1\}$ be the years with recorded births from recent to past. The merge starts from the most recent year t_n:

**Merging process for NDHS data**
1: **for** $t \in \{t_n, t_{n-1}, \cdots, t_1\}$ **do**
2:   **if** $t = t_n$ **then**
3:     Compute σ as explained in above. Compute CV= $\sigma/\log(r)^*_u$
4:     **if** CV < 0.05 **or** $t_n - t_{n-1} > 1$ **or** $t_{n+1} - t_n = 10$ **then**
5:       **stop** and move to the previous time point
6:     **else**
7:       Repeat step 3–5 based on births from t_n and t_{n-1}

The above procedures of computing sampling error and merging observation periods are performed for each NDHS.

1.3 TFR Data by Nepal Province

We compile the total fertility rate (TFR) by Nepal Province from NDHS 2001, 2006, 2011 and 2016 using the **R**-package `DHS.rates` [5, 6].

For the NDHS 2001, we adjust the microdata for all-women factor since the survey only included ever-married females and those who never got married by the time of the survey interview were not included [7]. We extract from the microdata of the "Household Member Recode" for NDHS 2001. The adjustment is done by multiplying the sample weight of each responded female by the all-women factor $AW_{p,a}$ for Province p and her age a by the time the survey was conducted.

Specifically, in each Nepal Province $p \in \{1, \cdots, 7\}$ (by matching the districts to province based on Table 1) for each reproductive age $a \in \{15, \cdots, 49\}$, we compute the total number of female household members $A_{p,a}$ for province p at age a:

$$A_{p,a} = \sum_{h=1}^{H_p} \mathbb{I}(v_{p,h} = \text{female})\mathbb{I}(y_{p,h} = \text{sleep at home})\mathbb{I}(\text{age}_{p,h} = a),$$

where H_p is the total number of household members in Nepal Province p, $v_{p,h}$ is the sex of the h-th household member in Province p, $y_{p,h}$ is whether the household member slept at home the day before the survey

interview conducted and $\text{age}_{p,h}$ is the age of the household member. Then we compute the number of ever-married female $E_{p,a}$ in Nepal Province p at age a:

$$E_{p,a} = \sum_{h=1}^{H_p} \mathbb{I}(v_{p,h} = \text{female})\mathbb{I}(y_{p,h} = \text{sleep at home})\mathbb{I}(\text{age}_{p,h} = a)\mathbb{I}(m_{p,h} = \text{ever married}),$$

where $m_{p,h}$ is the marital status and we select those household members who are ever-married. When there is no ever-married women for a certain age a in Province p, i.e. when $E_{p,a} = 0$, we merge the number of ever-married women with the number of one year older until the merged number is positive. Hence,

$$\begin{aligned} E^*_{p,a} &= E_{p,a} + E_{p,a+1}, \text{ if } E_{p,a} = 0, \\ E^*_{p,a} &= E^*_{p,a+1}. \end{aligned}$$

Subsequently, we merge $A_{p,a}$ using the same merged ages that we did for $E_{p,a}$ in order to obtain the corresponding $A^*_{p,a}$. Finally, the all-woman adjustment factor for Province p female age a is:

$$AW_{p,a} = \frac{A^*_{p,a}}{E^*_{p,a}}.$$

The TFR for the period beyond 2016 is based on the medium fertility scenario of population projections of 753 municipalities of Nepal, which was an update to the earlier projection [8]. We then aggregate the municipality births and women's years of exposure by province to generate the provincial TFR.

2 Model

2.1 Noations

Table 2 summarizes the notations and indexes used in this paper. $\mathcal{N}(\mu, \sigma^2)$ refers to a normal distribution with mean μ and variance σ^2. $t_3(\mu, \sigma^2)$ refers to a Student-*t* distribution with degrees of freedom 3, mean μ and variance σ^2. $\mathcal{U}(a, b)$ denotes a continuous uniform distribution with lower and upper bounds at a and b respectively.

2.2 Model for Sex Ratio at Birth by Nepal Province

The model is largely based on the model described in [9, 12]. In this study, we made a few modifications in the model to better address the data quality and availability of provincial SRB in Nepal. The outcome of interest $\Theta_{p,t}$, the SRB in Nepal Province p in year t is modeled as:

$$\begin{aligned} \Theta_{p,t} &= b\Phi_{p,t} + \delta_p \alpha_{p,t}, && (1) \\ \log(\Phi_{p,t}) &\sim \mathcal{N}(0, \sigma^2_\epsilon/(1-\rho^2)), \text{if } t = 1980, && (2) \\ \log(\Phi_{p,t}) &= \rho \log(\Phi_{p,t-1}) + \epsilon_{p,t}, \text{if } t \in \{1981, \cdots, 2050\}, && (3) \\ \epsilon_{p,t} &\overset{\text{i.i.d.}}{\sim} \mathcal{N}(0, \sigma^2_\epsilon). && (4) \end{aligned}$$

$b = 1.049$ is the SRB baseline level for the entire Nepal. The Nepal SRB baseline b is estimated based on national SRB observations from Nepal before reference year 1970 [10, 11]. $\Phi_{p,t}$ follows an AR(1) times series model on the log scale to capture the natural fluctuations of SRB within each province over time. $\rho = 0.9$ and $\sigma_\epsilon = 0.004$ based on previous study [10, 11]. For more detailed motivations of the setup for $\Phi_{p,t}$, please refer to [9, 10].

Symbol	Description
Index	
i	Indicator for the i-th SRB observation across all province-years, $i \in \{1, \ldots, 92\}$.
t	Indicator for year, $t \in \{1980, \ldots, 2050\}$.
p	Indicator for Nepal Province, $p \in \{1, \ldots, 7\}$.
Unknown Parameters	
$\Theta_{p,t}$	The model fitting for the true SRB for Nepal Province p in year t.
$\Phi_{p,t}$	The province-year-specific multiplier for capturing the natural fluctuation in SRB around the national baseline b for Nepal Province p in year t.
$\alpha_{p,t}$	The SRB imbalance for Nepal Province p in year t.
t_{0p}	The start year of the SRB inflation for Nepal Province p.
δ_p	The indicator of the presence ($\delta_p = 1$) or absence ($\delta_p = 0$) of SRB inflation in Nepal Province p.
ξ_p	The maximum level of the SRB inflation for Nepal Province p.
λ_{1p}	The period length for the stage of increase of the sex ratio transition for Nepal Province p.
λ_{2p}	The period length for the stage of stagnation of the sex ratio transition for Nepal Province p.
λ_{3p}	The period length for the stage of decrease back to national SRB baseline of the sex ratio transition for Nepal Province p.
Known Quantities	
r_i	The i-th SRB observation.
σ_i	The sampling error for the i-th SRB observation as computed in Section 1.2.
x_p	The year in which the total fertility rate (TFR) in Nepal Province p declines to 2.6 children per woman [9].
b	The baseline level of SRB for the whole Nepal [10], where $b = 1.049$.
ρ	The autoregressive indicator for $\Phi_{p,t}$, where $\rho = 0.9$ [10, 11].
σ_ϵ	The SD of distortion parameter for $\Phi_{p,t}$, where $\sigma_\epsilon = 0.004$ [10, 11].

Table 2: **Notation summary.**

Instead of estimating ρ and σ_ϵ, we use the estimated values from prior study [10, 11]. The rationale for this model setup is that ρ and σ_ϵ reflect the global experience of the temporal fluctuation under natural circumstances. These parameter were estimated based on all available SRB observations without risk of having SRB imbalance before reference year 1970 in [10, 11]. The studies were based on an extensive national SRB database and the estimates of the parameters ρ and σ_ϵ are robust. For Nepal, there are no observed provincial SRB before 1970 (the year in which the sex selection technology started to become available). Hence, all the SRB observations by Nepal province are at risk of SRB imbalance and these data points are considered not suitable to estimate ρ and σ_ϵ.

δ_p is the binary identifier of the sex ratio transition, following a Bernoulli distribution:

$$\begin{aligned} \delta_p|\pi_p &\sim \mathcal{B}(\pi_p), \text{ for } p \in \{1, \cdots, 7\}, &(5)\\ \text{logit}(\pi_p)|\mu_\pi, \sigma_\pi &\sim \mathcal{N}(\mu_\pi, \sigma_\pi^2), \text{ for } p \in \{1, \cdots, 7\}. &(6)\end{aligned}$$

The logit transformation ensures that the probability parameter π_p lies in the interval [0, 1]. The logit-transformed π_p follows a hierarchical normal distribution with a global mean and variance μ_π and σ_π^2.

$\alpha_{p,t}$ refers to the province-specific SRB imbalance process, and is modeled by a trapezoid function to represent the increase, stagnation, and decrease of the transition stages:

$$\begin{aligned} \alpha_{p,t} &= \begin{cases} (\xi_p/\lambda_{1p})(t-\gamma_p), & t_{0p} < t < t_{1p} \\ \xi_p, & t_{1p} < t < t_{2p} \\ \xi_c - (\xi_c/\lambda_{3p})(t - t_{2p}), & t_{2p} < t < t_{3p} \\ 0, & t < t_{0p} \text{ or } t > t_{3p} \end{cases} &(7)\\ t_{1p} &= t_{0p} + \lambda_{1p}, &(8)\\ t_{2p} &= t_{1p} + \lambda_{2p}, &(9)\\ t_{3p} &= t_{2p} + \lambda_{3p}, &(10)\\ t_{0p}|\sigma_{t0} &\sim t_3(x_p, \sigma_{t0}^2), \text{ for } p \in \{1, \cdots, 7\}. &(11)\end{aligned}$$

The start year of SRB inflation t_{0p} incorporates the fertility squeeze effect by using the data of total fertility rate (TFR). t_{0p} is modeled with a Student-t distribution with degrees of freedom 3 with mean at x_p which indicates the year in which the TFR in Nepal Province p declines to 2.6 [9]. We use the TFR 2.6 to determine the mean of the distribution for t_{0p} because this is the estimated TFR for which Nepal may start the sex ratio transition [9]. The low degrees of freedom for the Student-t distribution is needed to capture higher uncertainty at the tails for the distribution of t_{0p}. Please refer to [9] for more details on the model setups for $\alpha_{p,t}$.

2.3 Data quality model

r_i is the i-th observed SRB from province $p[i]$ in year $t[i]$, where i indexes all the SRB observations across provinces over time. r_i is assumed to follow a normal distribution on the log scale with mean at $\log(\Theta_{p[i],t[i]})$ (explained as above) and variance at σ_i^2:

$$\log(r_i)|\Theta_{p[i],t[i]} \sim \mathcal{N}(\log(\Theta_{p[i],t[i]}), \sigma_i^2), \text{ for } i \in \{1, \cdots, 92\}, \quad (12)$$

σ_i^2 is the sampling error variance for $\log(r_i)$ which reflects the uncertainty associated with log-scaled SRB observations due to survey sampling design. σ_i^2 is known and are calculated as explained in Section 1.2. We impute the sampling error variance for observations from 2011 Census as the median sampling error variance of all the other observations.

2.4 Priors

Informative priors Informative priors are assigned to province-level parameters related to the sex ratio transition: the maximum level of SRB inflation ξ_p, the period lengths for the stages of increase, stagnation and decrease as λ_{1p}, λ_{2p} and λ_{3p} respectively. The means of the prior distributions are from the systematic study [9] which modeled the sex ratio transition for multiple countries including Nepal. The standard deviations of the prior distribution is set such that the coefficient of variation (CV; defined as the ratio of mean to standard deviation) is 0.1. The informative priors could assist in modeling the sex ratio transition on the province level in Nepal by making use of the corresponding information on the national level. The usage of such informative priors is especially helpful in modeling subnational SRB imbalance in countries with great demographic heterogeneity [13, 14]. For $p \in \{1, \cdots, 7\}$:

$$\xi_p \sim \mathcal{N}(\mu_\xi, (\mu_\xi \text{CV})^2), \tag{13}$$
$$\lambda_{1p} \sim \mathcal{N}(\mu_{\lambda 1}, (\mu_{\lambda 1} \text{CV})^2), \tag{14}$$
$$\lambda_{2p} \sim \mathcal{N}(\mu_{\lambda 2}, (\mu_{\lambda 2} \text{CV})^2), \tag{15}$$
$$\lambda_{3p} \sim \mathcal{N}(\mu_{\lambda 3}, (\mu_{\lambda 3} \text{CV})^2), \tag{16}$$

where $\mu_\xi = 0.06$, $\mu_{\lambda 1} = 11.9$, $\mu_{\lambda 2} = 7.6$, $\mu_{\lambda 3} = 16.2$, and CV$= 0.1$.

Vague priors Vague priors are assigned to parameters related to the indicator for detecting the existence of a sex ratio transition and the SD of start year:

$$\text{inverse-logit}(\mu_\pi) \sim \mathcal{U}(0, 1), \tag{17}$$
$$\sigma_\pi \sim \mathcal{U}(0, 2), \tag{18}$$
$$\sigma_{t0} \sim \mathcal{U}(0, 10), \tag{19}$$

where $\mathcal{U}(a, b)$ indicates a continuous uniform distribution with lower and upper bounds at a and b respectively.

2.5 Statistical Computing

We obtained posterior samples of all the model parameters and hyper parameters using a Markov chain Monte Carlo (MCMC) algorithm, implemented in the open source softwares `R 3.6.1` [15] and `JAGS 4.3.0` [16], using `R`-packages **R2jags** [17] and **rjags** [18]. Results were obtained from 10 chains with a total number of 5,000 iterations in each chain, while the first 1,000 iterations were discarded as burn-in. After discarding burn-in iterations and proper thinning, the final posterior sample size for each parameter by combining all chains is 25,000. Convergence of the MCMC algorithm and the sufficiency of the number of samples obtained were checked through visual inspection of trace plots and convergence diagnostics of Gelman and Rubin [19], implemented in the **coda** `R`-package [20].

3 Model Validation

We assess the inflation model performance via two approaches: 1) out-of-sample validation; and 2) one-province simulation.

3.1 Out-of-Sample Validation

We leave out 20% of the data points since the data collection year 2016 instead of reference year, which has been used in assessing model performance for demographic indicators largely based on survey data [21, 22, 23, 24]. After leaving out data, we fit the model to the training data set, and obtain point estimates and credible intervals that would have been constructed based on available data set in the survey year selected.

We calculate median errors and median absolute errors for the left-out observations, where errors are defined as: $e_j = r_j - \widetilde{r}_j$, where $\widetilde{r}_j$ refers to the posterior median of the predictive distribution based on the training data set for the j-th left-out observation r_j. Coverage is given by $1/J \sum \mathbb{I}[r_j \geq l_j]\mathbb{I}[r_j \leq u_j]$, where J refers to the number of left-out observations, and l_j and u_j correspond to the lower and upper bounds of the 95% prediction interval for the j-th left-out observation r_j. The validation measures are calculated for 1000 sets of left-out observations, where each set consists one randomly selected left-out observation from each Nepal Province. The reported validation results are based on the mean of the outcomes from the 1000 sets of left-out observations.

For the point estimates based on full data set and training data set, errors for the true level of SRB are defined as $e(\Theta)_{p,t} = \widehat{\Theta}_{p,t} - \widetilde{\Theta}_{p,t}$, where $\widehat{\Theta}_{p,t}$ is the posterior median for Province p in year t based on the full data set, and $\widetilde{\Theta}_{p,t}$ is the posterior median for the same province-year based on the training data set. Similarly, the error for the sex ratio transition process with probability is defined as $e(\alpha\delta)_{p,t} = \widehat{\alpha}_{p,t}\widehat{\delta}_p - \widetilde{\alpha}_{p,t}\widetilde{\delta}_p$. Coverage is computed in a similar manner as for the left-out observations, based on the lower and upper bounds of the 95% credible interval of $\widetilde{\Theta}_{p,t}$ from the training data set.

3.2 One-Province Simulation

We assess the inflation model performance by one-province simulation. For each of the 7 Nepal Provinces, we consider all data points as test data and simulate the SRB using the posterior samples of the global parameters from the sex ratio transition model (obtained using the full dataset).

The g-th simulated SRB $\Theta_{p,t}^{(g)}$ for Province p in year t, and the g-th simulated SRB $\Theta_{p[j],t[j]}^{(g)}$ for the j-th left-out data point for Province $p[j]$ in year $t[j]$ are obtained as follows for $g \in \{1, \ldots, G\}$: $\Theta_{p,t}^{(g)} = b\Phi_{p,t}^{(g)} + \alpha_{p,t}^{(g)}\delta_p^{(g)}$, where $\Phi_{p[j],t[j]}^{(g)}$, $\alpha_{p[j],t[j]}^{(g)}$ and $\delta_p^{(g)}$ are simulated to refer to a "new" province, without taking into account any province-specific data, following the model specification for these parameters. $\alpha_{p,t}$ and δ_p are simulated using the posterior samples of all parameters and hyper-parameters related to them. After generating the simulated values, we calculate the same set of results as described in Section 3.1 on out-of-sample validation.

4 Validation and Simulation Results

Table 3 summarizes the results related to the left-out SRB observations for the out-of-sample validation exercise and the one-country simulation. Median errors and median absolute errors are very close to zero for left-out observations. The coverage of 95% and 80% prediction intervals are symmetrical and more conservative than expected. The wider-than-expected prediction interval for left-out observations are mainly due to the greater uncertainty associated in more recent observations. The proportions of observations that fall below the prediction intervals constructed based on the one-country simulation are reasonable, given that the average number of observations fall below falling outside their respective bounds is at most 1.1.

Table 4 shows results for the comparison between model estimates obtained based on the full dataset and based on the training set for the out-of-sample validation exercise. We look at the model estimates for the true SRB $\Theta_{p,t}$ and the inflation process with country-specific probability $\delta_p\alpha_{p,t}$. Median errors and the median absolute errors are close to zero.

In summary, the validation results indicate reasonably good calibrations and predicting power of the inflation model with conservative credible intervals.

**Inflation Model**	**Validation Out-of-Sample**	**Simulation**
# Province in test dataset	7	7
Median error	0.008	0.006
Median absolute error	0.036	0.048
Below 95% prediction interval (%)	0.0	5.5 (0.4)
Above 95% prediction interval (%)	0.0	9.6 (0.7)
**Expected (%)**	**2.5**	**2.5**
Below 80% prediction interval (%)	0.0	6.5 (0.5)
Above 80% prediction interval (%)	4.9	15.3 (1.1)
**Expected (%)**	**10**	**10**

Table 3: **Validation and simulation results for left-out SRB observations.** Error is defined as the difference between a left-out SRB observation and the posterior median of its predictive distribution. SRB observations with data collection year since 2016 are left out. Numbers in the parentheses after the proportions indicate the average number of left-out observations fall below or above their respective 95% and 80% prediction intervals.

**Inflation Model Validation**		$\boldsymbol{\Theta_{p,t}}$			$\boldsymbol{\delta_p \alpha_{p,t}}$	
**(Out-of-Sample)**	**1995**	**2005**	**2015**	**1995**	**2005**	**2015**
Median error	0.001	0.001	0.000	0.000	0.000	0.000
Median absolute error	0.001	0.002	0.004	0.000	0.000	0.005
Below 95% credible interval (%)	0.0	0.0	0.0	0.0	0.0	0.0
Above 95% credible interval (%)	0.0	0.0	0.0	0.0	0.0	0.0
**Expected (%)**	**≤2.5**	**≤2.5**	**≤2.5**	**≤2.5**	**≤2.5**	**≤2.5**
Below 80% credible interval (%)	0.0	0.0	0.0	0.0	0.0	0.0
Above 80% credible interval (%)	0.0	0.0	0.0	0.0	0.0	0.0
**Expected (%)**	**≤10**	**≤10**	**≤10**	**≤10**	**≤10**	**≤10**

Table 4: **Validation results for estimates based on training set.** Error is defined as the differences between a model estimate (i.e. $\Theta_{p,t}$ or $\delta_p \alpha_{p,t}$) based on full dataset and training set. The proportions refer to the proportions (%) of countries in which the median estimates based on the full dataset fall below or above their respective 95% and 80% credible intervals based on the training set.

5 Sensitivity analyses

5.1 Analysis for start year distribution

We assessed the choice of distribution of the start year of SRB inflation (Section 2.2, Eq. 11) and its effect on the province-specific start year parameter t_{0p} and final outcome of interest SRB $\Theta_{p,t}$. We conducted an alternative approach to estimate the start year parameter t_{0p}:

$$\begin{aligned} t_{0p}|\sigma_{t0} &\sim t(x_p, \sigma_{t0}^2, \nu), \text{ for } p \in \{1, \cdots, 7\}, \\ \nu &\sim \mathcal{U}(1, 50), \end{aligned}$$

where $\mathcal{U}(a, b)$ indicates a continuous uniform distribution with lower and upper bounds at a and b respectively. Comparing with Eq. 11, instead of fixing the degrees of freedom of the Student-*t* distribution at 3 (i.e. $\nu = 3$), we assign a uniform prior to it.

The start year parameters t_{0p} are not sensitive to the choice of ν as shown in Table 5. The median estimates of t_{0p} are the same in most provinces between the two model assumptions on ν. The 95% credible intervals are also very similar to each other for all provinces.

The final outcome of interest SRB $\Theta_{p,t}$ is also not sensitive to the choice of ν, as shown in Table 6. The average difference in $\Theta_{p,t}$ over the full period 1980–2050 between $\nu = 3$ and $\nu \sim \mathcal{U}(1, 50)$ are not statistically significantly different from zero across all provinces. The median differences are very close to zero as well. The same conclusion can be drawn for the estimation period 1980–2016 and the projection period 2016–2050.

Figure 1 illustrates the comparison of the model fittings for SRB for the seven provinces based on $\nu = 3$ and $\nu \sim \mathcal{U}(1, 50)$. The median SRB and 95% credible intervals are almost identical for most provinces. Most of the observable differences (none is statistically significantly different from zero) in the median SRB are during the projection period after 2016.

t_{0p}	$\nu = 3$ (reference level)	$\nu \sim \mathcal{U}(1, 50)$
$p = 1$	2007 (1994, 2028)	2006 (1996, 2025)
$p = 2$	2018 (1999, 2040)	2018 (2001, 2035)
$p = 3$	2004 (1989, 2026)	2004 (1991, 2022)
$p = 4$	2007 (1976, 2029)	2008 (1979, 2026)
$p = 5$	2001 (1993, 2023)	2002 (1994, 2021)
$p = 6$	2013 (1989, 2036)	2013 (1993, 2031)
$p = 7$	2006 (1992, 2029)	2006 (1993, 2025)

Table 5: **Sensitivity analysis for t_{0p}.** Median estimates are numbers above brackets. 95% credible intervals are in brackets.

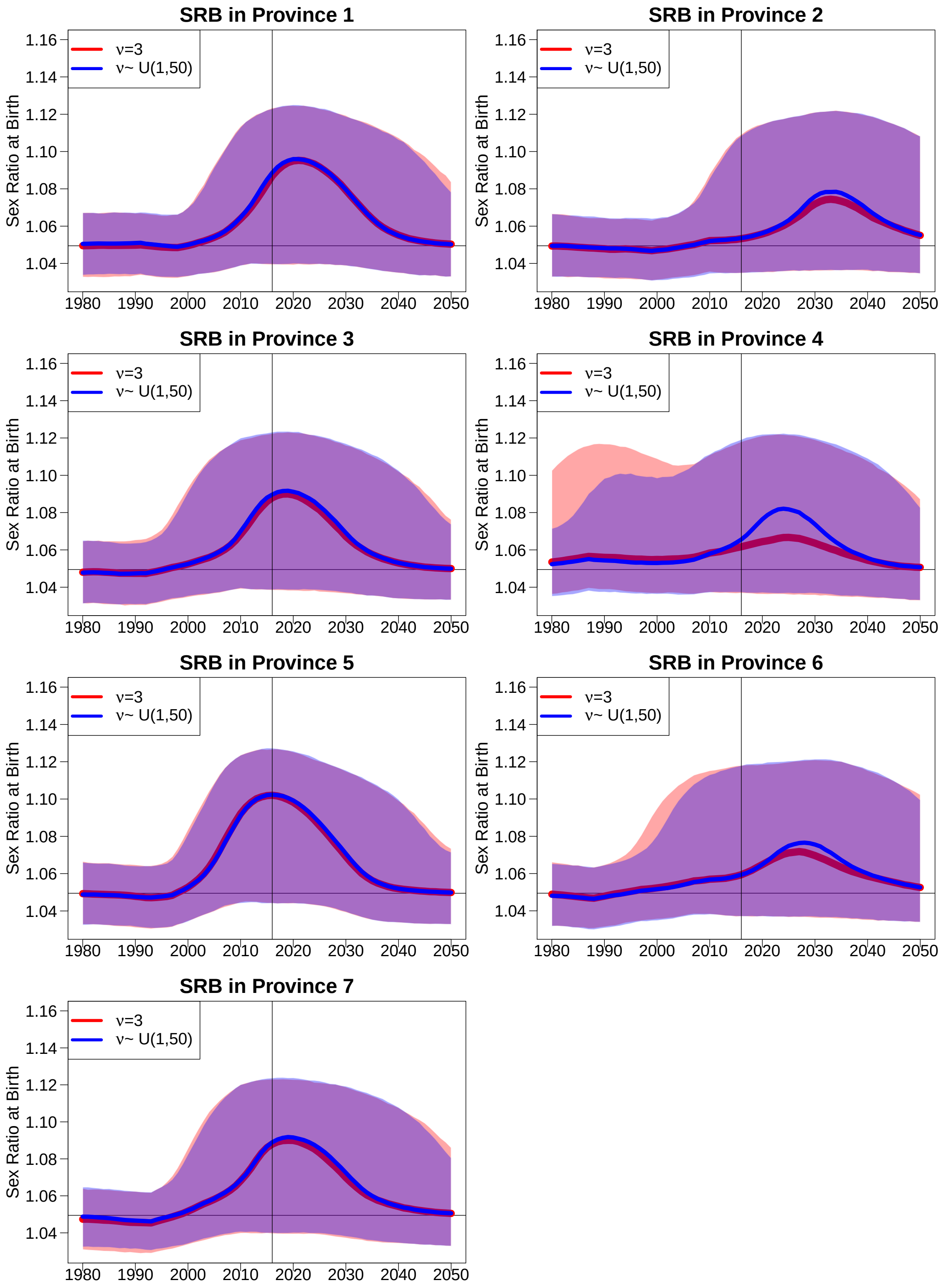

Figure 1: **SRB model results based on different start year distributions.** The median and 95% credible intervals of the province-specific SRB are in curves and shades. Results are differentiated by colors based on different start year distributions (Section 2.2, Eq. 11). The horizontal line refers to the SRB baseline for the whole Nepal at 1.049 [10]. Vertical line indicates the year 2016, which marks the start of projection period.

$\Theta_{p,t}$ difference	$\nu = 3$ (reference level)	$\nu \sim \mathcal{U}(1, 50)$ (1980–2050)	(1980–2016)	(2016–2050)
$p = 1$	–	0.0004	0.0006	0.0001
	–	(-0.0221, 0.0221)	(-0.0221, 0.0231)	(-0.0355, 0.0356)
$p = 2$	–	0.0004	0.0001	0.0003
	–	(-0.0223, 0.0227)	(-0.0167, 0.0164)	(-0.0387, 0.0391)
$p = 3$	–	0.0003	0.0003	0.0005
	–	(-0.0224, 0.0228)	(-0.0281, 0.0281)	(-0.0346, 0.0352)
$p = 4$	–	-0.0003	-0.0019	0.0010
	–	(-0.0230, 0.0222)	(-0.0362, 0.0277)	(-0.0375, 0.0385)
$p = 5$	–	0.0001	-0.0003	0.0005
	–	(-0.0203, 0.0200)	(-0.0258, 0.0248)	(-0.0378, 0.0383)
$p = 6$	–	-0.0000	-0.0006	0.0005
	–	(-0.0224, 0.0223)	(-0.0287, 0.0245)	(-0.0378, 0.0383)
$p = 7$	–	0.0006	0.0005	0.0006
	–	(-0.0214, 0.0226)	(-0.0264, 0.0270)	(-0.0347, 0.0356)

Table 6: **Sensitivity analysis for $\Theta_{p,t}$ with different start year distributions.** Median estimates are numbers above brackets. 95% credible intervals are in brackets (all include zero, i.e. not statistically significantly different from zero). Results are the average difference in $\Theta_{p,t}$ during whole period 1980–2050, estimation period 1980–2016, and projection period 2016–2050 when $\nu \sim \mathcal{U}(1, 50)$ and when $\nu = 3$.

5.2 Analysis for sex ratio transition variances

We assessed the effect of the sex ratio transition variances, by setting the CV value (explained in Section 2.4, Eq. 13–Eq. 16), on the estimates and 95% credible bounds for the outcome of interest $\Theta_{p,t}$ and parameters that are related to the sex ratio transition.

Table 7 shows the average difference in $\Theta_{p,t}$, the SRB, over the period of 1980–2050 between other model assumptions (i.e. CV$= 0.2$ and CV$= 0.5$) and the reference level (i.e. the final model setting with CV$= 0.1$). Across all seven provinces with $p \in \{1, ..., 7\}$, all differences are close to zero and none of the differences are statistically significantly different from zero. Similarly, when looking at the differences in $\Theta_{p,t}$ during the estimation period 1980–2016 in Table 8, and the differences during the projection period 2016–2050 in Table 9, they are all close to zero and none of the differences are statistically significantly different from zero.

Figure 2 illustrates the SRB model results based on different CV values assigned to the informative priors (explained in Section 2.4). The median estimates and projections of SRB are very similar when CV is 0.1 and 0.2. When CV$= 0.5$, the projected sex ratio transition is milder and the projected SRB is less deviated from the national baseline for all provinces except for Province 5. The deviation become more pronounced after 2016, which is the start of the projection period. As the CV increases, the credible intervals become wider.

Table 10, 11, 12, and 13 present the model results of the province-specific parameters ξ_p (maximum SRB inflation), and λ_{1p}, λ_{2p}, λ_{3p} (period lengths of increase, stagnation, and decrease of sex ratio transition process) based on different CV's used in their prior distributions. In general, the median estimates for all parameters based on different CV's are very similar. The 95% credible intervals of these parameters, however, increase as the CV increases. This is mainly because few data points of Nepal provincial SRB indicate a past or ongoing sex ratio transition. Consequently, the model suggest that the sex ratio transition process will mostly happen in the projection period.

$\Theta_{p,t}$ difference (1980–2050)	CV $= 0.1$ (reference level)	CV $= 0.2$	CV $= 0.5$
$p = 1$	–	0.0002	0.0008
	–	(-0.0214, 0.0223)	(-0.0213, 0.0299)
$p = 2$	–	-0.0004	-0.0006
	–	(-0.0218, 0.0224)	(-0.022, 0.0274)
$p = 3$	–	0.0003	0.0003
	–	(-0.0212, 0.0232)	(-0.0216, 0.0303)
$p = 4$	–	0.000	-0.0005
	–	(-0.0215, 0.0229)	(-0.0223, 0.0288)
$p = 5$	–	0.0003	0.0032
	–	(-0.0193, 0.0200)	(-0.019, 0.0299)
$p = 6$	–	-0.0003	-0.0007
	–	(-0.0217, 0.0225)	(-0.0221, 0.0287)
$p = 7$	–	-0.000	0.0004
	–	(-0.0214, 0.0223)	(-0.0216, 0.0292)

Table 7: **Sensitivity analysis for $\Theta_{p,t}$, 1980–2050.** Median estimates are numbers above brackets. 95% credible intervals are in brackets (all include zero, i.e. not statistically significantly different from zero). Results are the average difference in $\Theta_{p,t}$ during 1980–2050 when CV$\neq 0.1$ and when CV$= 0.1$.

$\Theta_{p,t}$ difference (1980–2016)	CV $= 0.1$ (reference level)	CV $= 0.2$	CV $= 0.5$
$p = 1$	–	-0.0001	0.0008
	–	(-0.0232, 0.0234)	(-0.0227, 0.0257)
$p = 2$	–	-0.0005	-0.0003
	–	(-0.0169, 0.0167)	(-0.0168, 0.0174)
$p = 3$	–	0.0003	-0.0000
	–	(-0.0278, 0.0289)	(-0.0283, 0.0312)
$p = 4$	–	0.0001	-0.0010
	–	(-0.0348, 0.0367)	(-0.0357, 0.0424)
$p = 5$	–	0.0002	0.0038
	–	(-0.0259, 0.0261)	(-0.0246, 0.0326)
$p = 6$	–	-0.0004	-0.0004
	–	(-0.0287, 0.0283)	(-0.0285, 0.0291)
$p = 7$	–	-0.0002	-0.0003
	–	(-0.0274, 0.0275)	(-0.0275, 0.0302)

Table 8: **Sensitivity analysis for $\Theta_{p,t}$, 1980–2016.** Median estimates are numbers above brackets. 95% credible intervals are in brackets (all include zero, i.e. not statistically significantly different from zero). Results are the average difference in $\Theta_{p,t}$ during 1980–2016 when CV$\neq 0.1$ and when CV$= 0.1$.

$\Theta_{p,t}$ difference (2016–2050)	CV $= 0.1$ (reference level)	CV $= 0.2$	CV $= 0.5$
$p = 1$	–	0.0001	0.0003
	–	(-0.0358, 0.0376)	(-0.0362, 0.0549)
$p = 2$	–	-0.0001	-0.0004
	–	(-0.039, 0.0422)	(-0.0391, 0.0546)
$p = 3$	–	0.0001	0.0000
	–	(-0.0348, 0.0381)	(-0.0353, 0.0540)
$p = 4$	–	-0.0001	-0.0002
	–	(-0.0381, 0.0411)	(-0.0383, 0.0566)
$p = 5$	–	0.0003	0.0024
	–	(-0.0295, 0.0319)	(-0.031, 0.0508)
$p = 6$	–	-0.0002	-0.0005
	–	(-0.038, 0.0415)	(-0.0385, 0.0559)
$p = 7$	–	-0.0001	0.0002
	–	(-0.0353, 0.0378)	(-0.0358, 0.0544)

Table 9: **Sensitivity analysis for $\Theta_{p,t}$, 2016–2050.** Median estimates are numbers above brackets. 95% credible intervals are in brackets (all include zero, i.e. not statistically significantly different from zero). Results are the average difference in $\Theta_{p,t}$ during 2016–2050 when CV$\neq 0.1$ and when CV$= 0.1$.

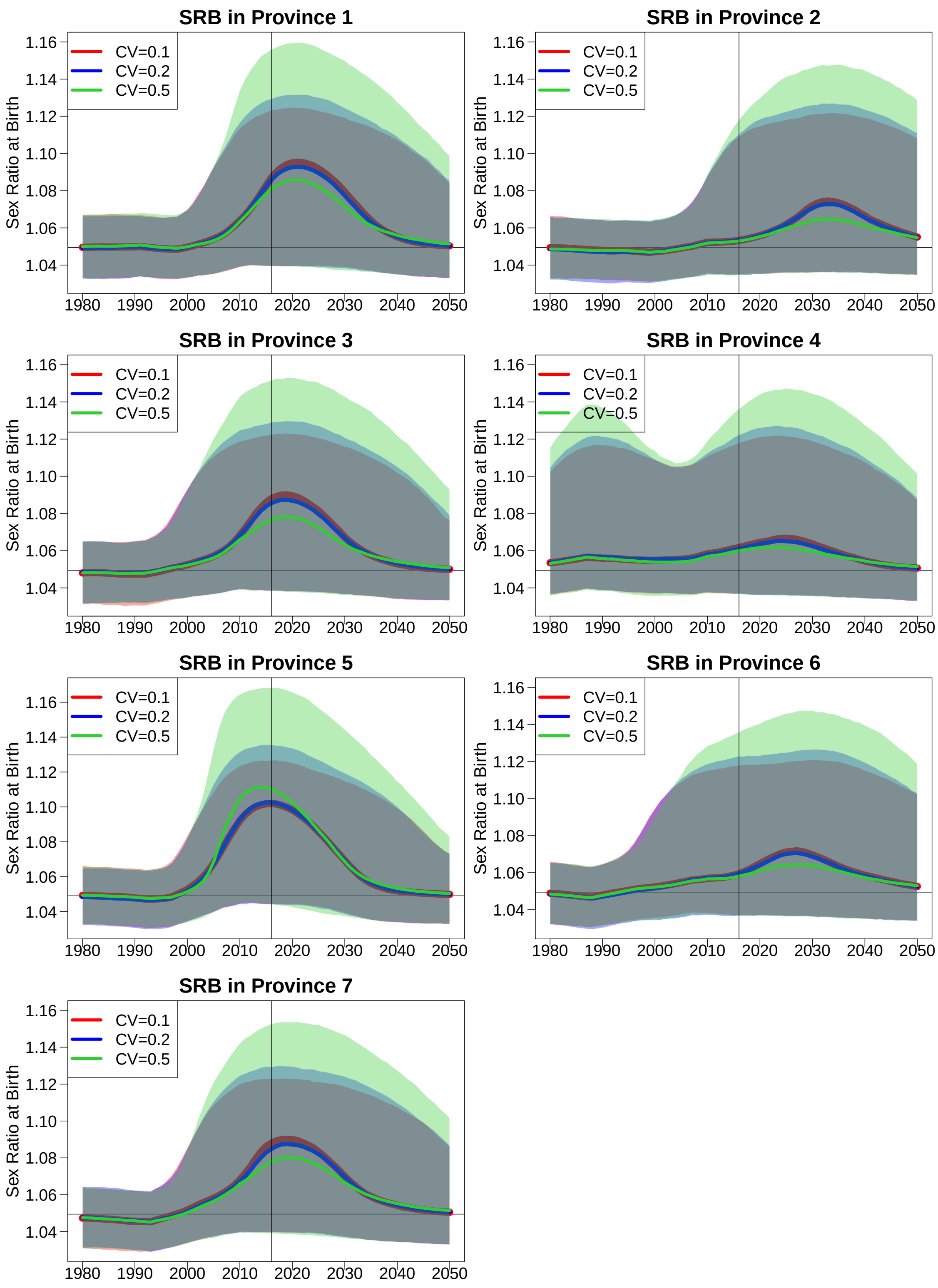

Figure 2: **SRB model results based on different CV.** The median and 95% credible intervals of the province-specific SRB are in curves and shades. Results are differentiated by colors based on different CV values assigned to priors (Section 2.4). The horizontal line refers to the SRB baseline for the whole Nepal at 1.049 [10]. Vertical line indicates the year 2016, which marks the start of projection period.

ξ_p	CV = 0.1	CV = 0.2	CV = 0.5
$p = 1$	0.058<br>(0.047, 0.069)	0.059<br>(0.036, 0.081)	0.063<br>(0.013, 0.117)
$p = 2$	0.058<br>(0.047, 0.069)	0.058<br>(0.035, 0.080)	0.059<br>(0.009, 0.114)
$p = 3$	0.058<br>(0.047, 0.069)	0.058<br>(0.036, 0.080)	0.061<br>(0.012, 0.114)
$p = 4$	0.058<br>(0.047, 0.069)	0.058<br>(0.036, 0.081)	0.060<br>(0.011, 0.114)
$p = 5$	0.058<br>(0.047, 0.07)	0.061<br>(0.038, 0.083)	0.074<br>(0.021, 0.124)
$p = 6$	0.058<br>(0.047, 0.069)	0.058<br>(0.036, 0.080)	0.059<br>(0.010, 0.113)
$p = 7$	0.058<br>(0.047, 0.069)	0.059<br>(0.036, 0.081)	0.063<br>(0.013, 0.115)

Table 10: **Sensitivity analysis for ξ_p.** Median estimates are numbers above brackets. 95% credible intervals are in brackets.

λ_{1p}	CV = 0.1	CV = 0.2	CV = 0.5
$p = 1$	11.82<br>(9.48, 14.15)	11.70<br>(7.06, 16.31)	11.23<br>(1.46, 22.67)
$p = 2$	11.85<br>(9.51, 14.18)	11.86<br>(7.27, 16.49)	12.01<br>(1.89, 23.60)
$p = 3$	11.83<br>(9.50, 14.13)	11.75<br>(7.14, 16.40)	11.67<br>(1.78, 23.12)
$p = 4$	11.83<br>(9.53, 14.15)	11.83<br>(7.19, 16.51)	11.79<br>(1.79, 23.32)
$p = 5$	11.75<br>(9.41, 14.09)	11.42<br>(6.71, 16.07)	9.61<br>(0.95, 21.49)
$p = 6$	11.84<br>(9.50, 14.16)	11.80<br>(7.17, 16.45)	11.85<br>(1.90, 23.63)
$p = 7$	11.80<br>(9.49, 14.12)	11.72<br>(7.10, 16.4)	11.40<br>(1.57, 22.91)

Table 11: **Sensitivity analysis for λ_{1p}.** Median estimates are numbers above brackets. 95% credible intervals are in brackets.

λ_{2p}	CV = 0.1	CV = 0.2	CV = 0.5
$p = 1$	7.56<br>(6.08, 9.06)	7.56<br>(4.58, 10.51)	7.70<br>(1.25, 15.10)
$p = 2$	7.57<br>(6.08, 9.05)	7.58<br>(4.57, 10.56)	7.67<br>(1.19, 15.11)
$p = 3$	7.59<br>(6.09, 9.06)	7.57<br>(4.59, 10.55)	7.71<br>(1.23, 15.06)
$p = 4$	7.57<br>(6.11, 9.06)	7.57<br>(4.56, 10.58)	7.60<br>(1.21, 14.93)
$p = 5$	7.57<br>(6.11, 9.08)	7.59<br>(4.64, 10.54)	7.67<br>(1.31, 14.99)
$p = 6$	7.57<br>(6.08, 9.05)	7.58<br>(4.60, 10.53)	7.71<br>(1.29, 15.00)
$p = 7$	7.57<br>(6.08, 9.06)	7.59<br>(4.60, 10.5)	7.66<br>(1.22, 15.01)

Table 12: **Sensitivity analysis for λ_{2p}.** Median estimates are numbers above brackets. 95% credible intervals are in brackets.

λ_{3p}	CV = 0.1	CV = 0.2	CV = 0.5
$p = 1$	16.17<br>(12.97, 19.35)	16.18<br>(9.82, 22.51)	16.39<br>(2.69, 32.27)
$p = 2$	16.16<br>(13.00, 19.33)	16.20<br>(9.84, 22.64)	16.39<br>(2.70, 31.9)
$p = 3$	16.17<br>(12.99, 19.40)	16.19<br>(9.86, 22.63)	16.34<br>(2.65, 31.86)
$p = 4$	16.19<br>(13.00, 19.39)	16.14<br>(9.79, 22.5)	16.52<br>(2.69, 32.41)
$p = 5$	16.15<br>(12.96, 19.36)	16.12<br>(9.82, 22.54)	16.36<br>(2.70, 32.11)
$p = 6$	16.17<br>(13.00, 19.33)	16.20<br>(9.83, 22.51)	16.49<br>(2.66, 31.81)
$p = 7$	16.19<br>(12.98, 19.32)	16.15<br>(9.83, 22.52)	16.47<br>(2.77, 32.25)

Table 13: **Sensitivity analysis for λ_{3p}.** Median estimates are numbers above brackets. 95% credible intervals are in brackets.

5.3 Analysis for time series model

We assessed the model choice for $\Phi_{p,t}$, the province-year-specific factor that captures the year-by-year natural fluctuation within each province. We used AR(1) to model $\Phi_{p,t}$ (Eq. 2–Eq. 4). As explained in Section 2.2, we fixed AR(1) parameters. Hence, in order to fully assess the model choice for $\Phi_{p,t}$, we compare the $\Phi_{p,t}$ based on AR(1) and AR(2) by modeling all parameters related to the time series model. The AR(1) model with ρ and σ_ϵ not fixed:

$$
\begin{aligned}
\log(\Phi_{p,t}) &\sim \mathcal{N}(0, \sigma_\epsilon^2/(1-\rho^2)), \text{if } t = 1980, \\
\log(\Phi_{p,t}) &= \rho \log(\Phi_{p,t-1}) + \epsilon_{p,t}, \text{if } t \in \{1981, \cdots, 2050\}, \\
\epsilon_{p,t} &\overset{\text{i.i.d.}}{\sim} \mathcal{N}(0, \sigma_\epsilon^2), \\
\rho &\sim \mathcal{U}(0, 1), \\
\sigma_\epsilon &\sim \mathcal{U}(0, 0.05).
\end{aligned}
$$

The AR(2) model for $\Phi_{p,t}$ with ρ_1, ρ_2, and σ_ϵ not fixed:

$$
\begin{aligned}
\log(\Phi_{p,t}) &\sim \mathcal{N}\left(0, \frac{\sigma_\epsilon^2}{(1+\rho_2)/(1-\rho_2)((1-\rho_2)^2-\rho_1^2)}\right), \text{if } t = 1980, \\
\log(\Phi_{p,t}) &\sim \mathcal{N}\left(\frac{\rho_1}{1-\rho_2}\log(\Phi_{p,t-1}), \frac{\sigma_\epsilon^2}{1-\rho_2^2}\right), \text{if } t = 1981, \\
\log(\Phi_{p,t}) &= \rho_1 \log(\Phi_{p,t-1}) + \rho_2 \log(\Phi_{p,t-2}) + \epsilon_{p,t}, \text{if } t \in \{1982, \cdots, 2050\}, \\
\epsilon_{p,t} &\overset{\text{i.i.d.}}{\sim} \mathcal{N}(0, \sigma_\epsilon^2), \\
\rho_2 &\sim \mathcal{U}(0, 1), \\
\rho_1 &\sim \mathcal{U}(-|1-\rho_2|, |1-\rho_2|), \\
\sigma_\epsilon &\sim \mathcal{U}(0, 0.05),
\end{aligned}
$$

where for causality the roots of the AR(2) polynomial function $g(z) = 1 - \rho_1 z - \rho_2 z^2$ all lie within the unit circle.

Figure 3 illustrates the modeling fittings for $\Phi_{p,t}$, based on (i) AR(1) model and fixing ρ and σ_ϵ; (ii) AR(1) model and not fixing ρ and σ_ϵ; and (iii) AR(2) model and not fixing ρ_1, ρ_2, and σ_ϵ. In general, the model results for $\Phi_{p,t}$ based on settings of (ii) and (iii) are very similar in both median estimates and 95% credible intervals. Hence, given that $\Phi_{p,t}$ is not sensitive to the choice of AR(1) or AR(2), if all parameters related to $\Phi_{p,t}$ were estimated, we use the simpler model AR(1).

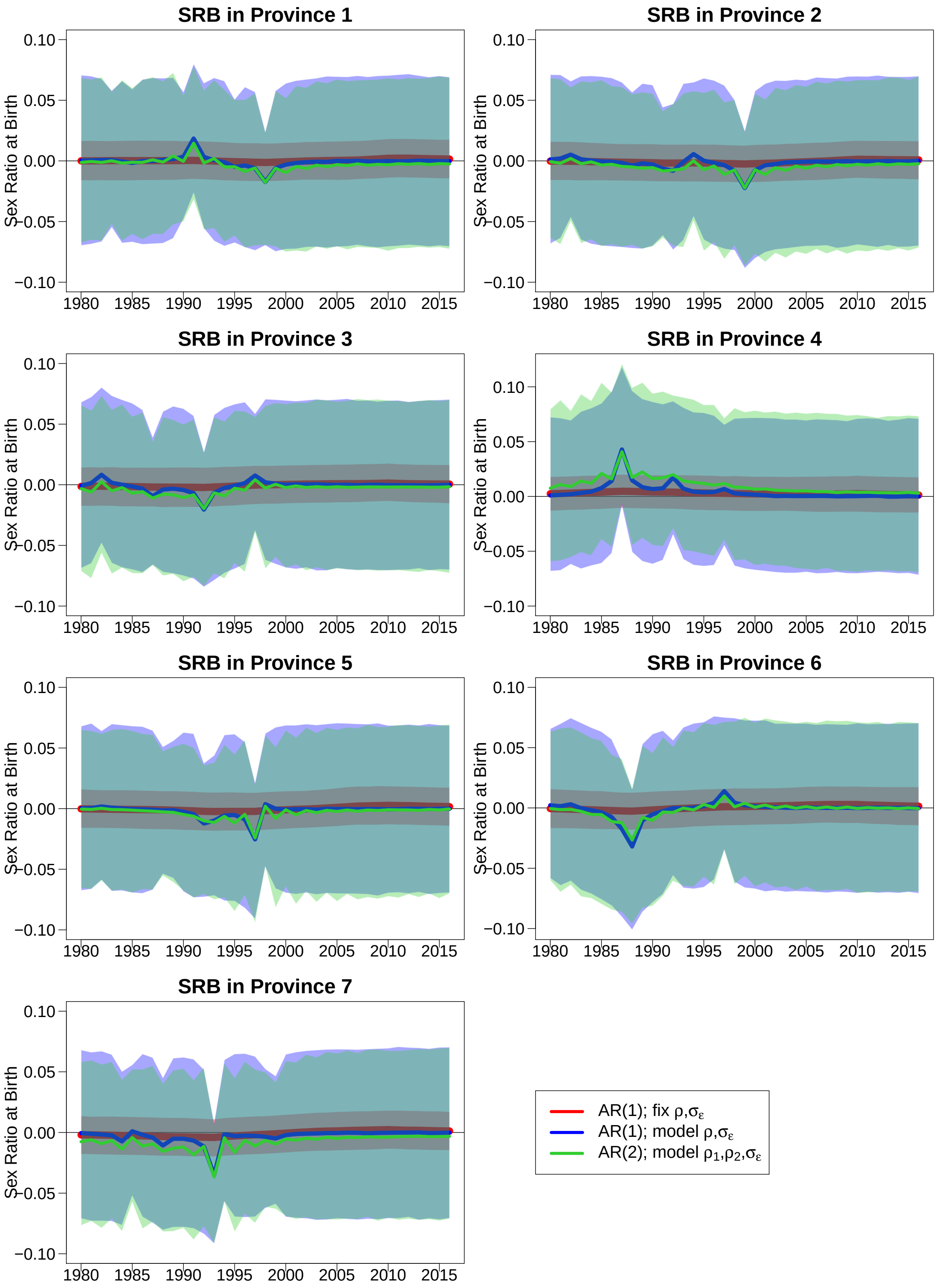

Figure 3: $\Phi_{p,t}$ **model results based on AR(1) and AR(2).** The median and 95% credible intervals of the province-specific SRB are in curves and shades.

6 Supplementary Figures

Figure 4: **SRB estimates and projections by Nepal Province, 1980–2050.** The red line and shades are the median and 95% credible intervals of the province-specific SRB. The green horizontal line refers to the SRB baseline for the whole Nepal at 1.049 [10]. SRB observations are displayed with dots and observations are connected with lines when obtained from the same source. Shaded areas around observation series represent the sampling variability in the series (quantified by two times the sampling standard errors). The blue squared dots are total fertility rate (TFR) extracted from NDHS. The median estimates of start year and end year of SRB inflation are indicated by vertical lines. The TFR value in the start year is shown.

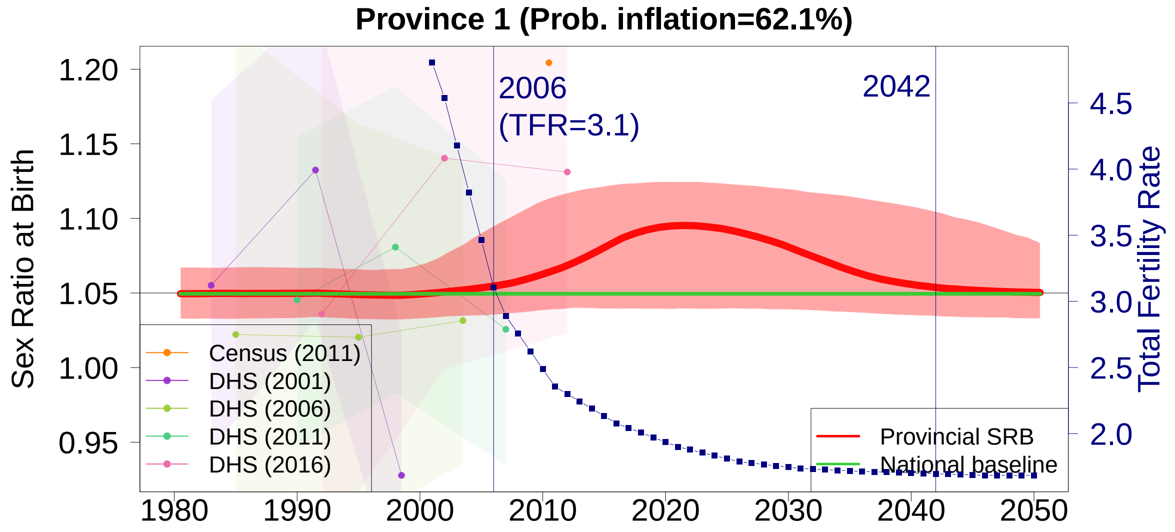

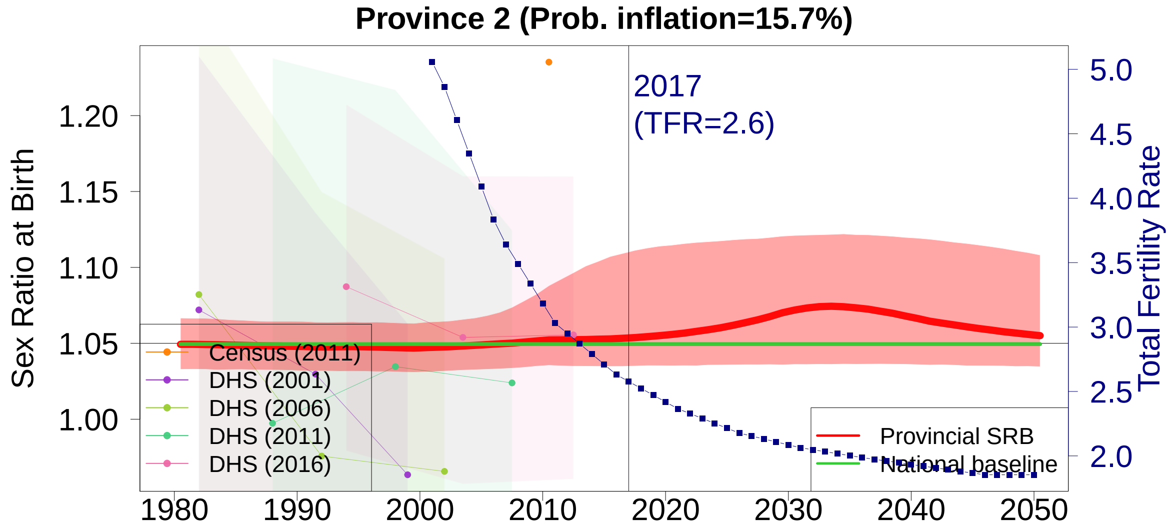

Continued on next page.

Figure 4 – continued from previous page.

Province 3 (Prob. inflation=63.3%)

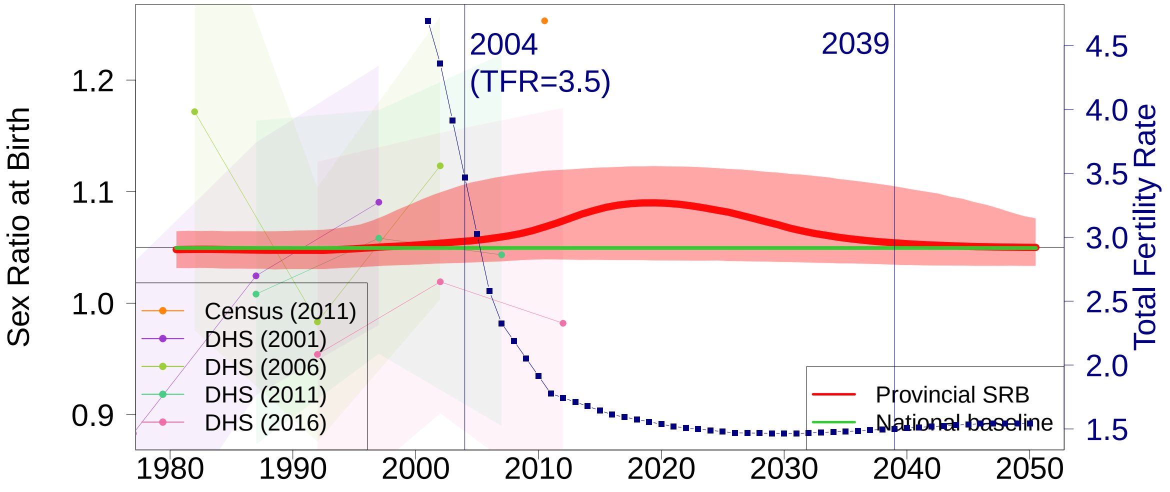

Province 4 (Prob. inflation=55.2%)

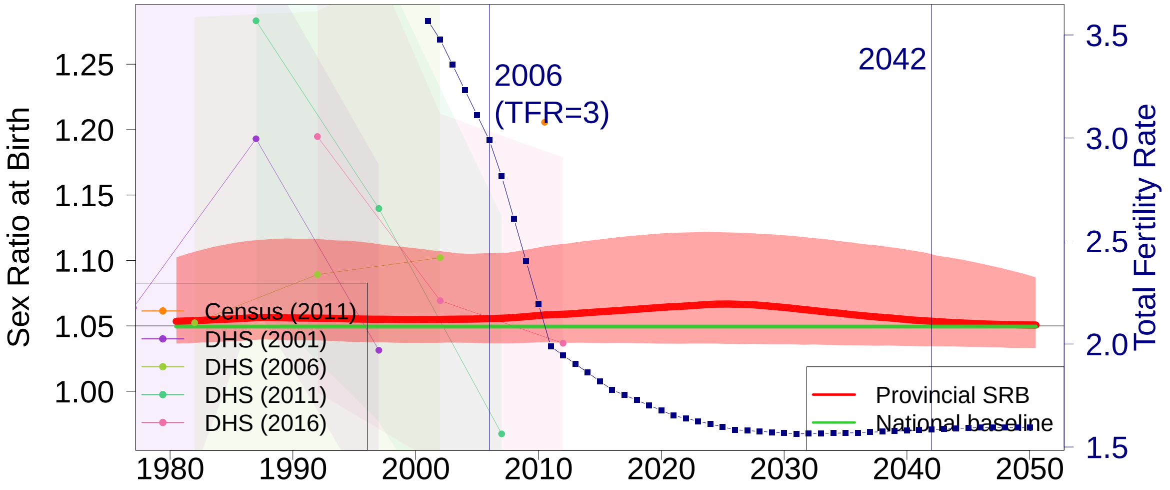

Province 5 (Prob. inflation=81.1%)

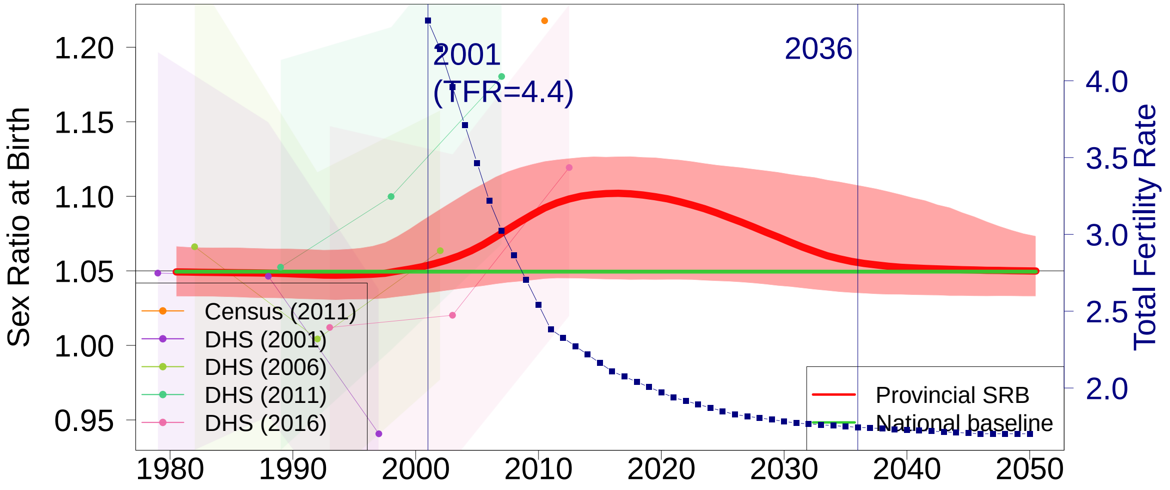

Continued on next page.

Figure 4 – continued from previous page.

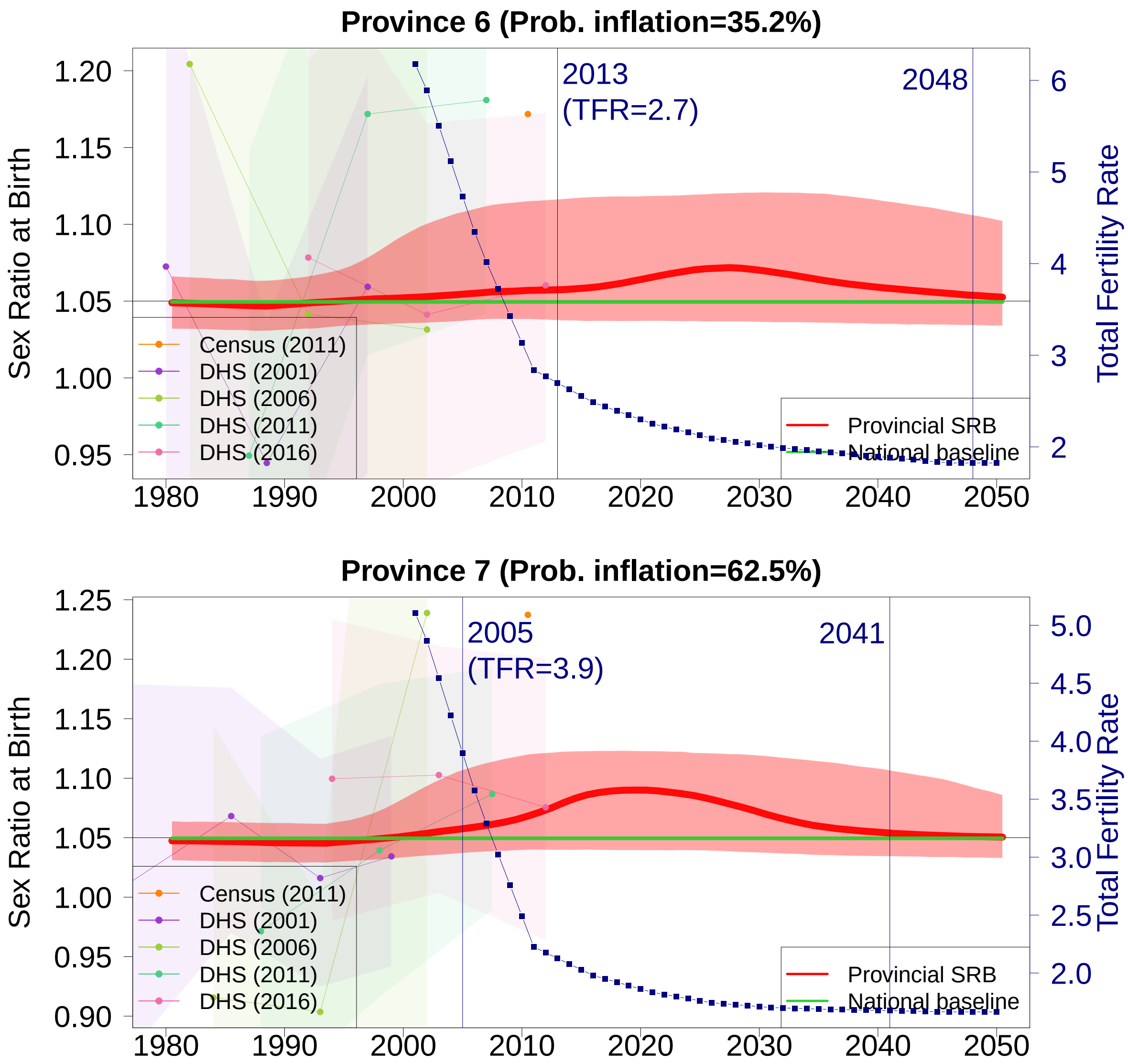

The end of Figure 4.

References

[1] Efron B, Gong G. A leisurely look at the bootstrap, the jackknife, and cross-validation. The American Statistician. 1983;37(1):36–48.

[2] Efron B, Tibshirani RJ. An introduction to the bootstrap. In: An introduction to the bootstrap. CHAPMAN & HALLI/CRC; 1994. .

[3] International ICF. Demographic and Health Survey Sampling and Household Listing Manual. Calverton, Maryland, U.S.A.: MEASURE DHS; 2012. p. 78–79. Available from: https://dhsprogram.com/pubs/pdf/DHSM4/DHS6_Sampling_Manual_Sept2012_DHSM4.pdf.

[4] Pedersen J, Liu J. Child mortality estimation: appropriate time periods for child mortality estimates from full birth histories. PLoS medicine. 2012;9(8).

[5] Elkasabi M. DHS.rates: Calculates Demographic Indicators; 2019.

[6] Elkasabi M. Calculating fertility and childhood mortality rates from survey data using the DHS.rates R package. PLOS ONE. 2019;14(5):1–22. Available from: 10.1371/journal.pone.0216403.

[7] Rutstein SO, Demographic and Health Surveys, ORC Macro. Guide to DHS statistics; 2006. Available from: http://citeseerx.ist.psu.edu/viewdoc/download?doi=10.1.1.431.8235&rep=rep1&type=pdf.

[8] KC S, Speringer M, Thapa A, Khanal MN. Projecting Nepal's Demographic Future-How to deal with spatial and demographic heterogeneity. 2016.

[9] Chao F, Gerland P, Cook AR, Alkema L. Global estimation and scenario-based projections of sex ratio at birth and missing female births using a Bayesian hierarchical time series mixture model. The Annals of Applied Statistics. 2021;15(3):1499–1528.

[10] Chao F, Gerland P, Cook AR, Alkema L. Systematic assessment of the sex ratio at birth for all countries and estimation of national imbalances and regional reference levels. Proceedings of the National Academy of Sciences. 2019;116(19):9303–9311.

[11] Chao F, Gerland P, Cook AR, Alkema L. Web Appendix Systematic assessment of the sex ratio at birth for all countries and estimation of national imbalances and regional reference levels. 2019. DOI: 10.6084/m9.figshare.12442373. Available at https://www.pnas.org/content/pnas/suppl/2019/04/10/1812593116.DCSupplemental/pnas.1812593116.sapp.pdf.

[12] Chao F, Gerland P, Cook AR, Guilmoto CZ, Alkema L. Projecting sex imbalances at birth at global, regional and national levels from 2021 to 2100: scenario-based Bayesian probabilistic projections of the sex ratio at birth and missing female births based on 3.26 billion birth records. BMJ Global Health. 2021;6(8):e005516.

[13] Chao F, Guilmoto CZ, Ombao H. Sex ratio at birth in Vietnam among six subnational regions during 1980–2050, estimation and probabilistic projection using a Bayesian hierarchical time series model with 2.9 million birth records. PLoS ONE. 2021;16(7):e0253721.

[14] Chao F, Wazir MA, Ombao H. Levels and trends in sex ratio at birth in provinces of Pakistan from 1980 to 2020 with scenario-based missing female birth projections to 2050: a Bayesian modeling approach. SocArXiv. 2021. DOI: 10.31235/osf.io/5n3y8. Available at https://osf.io/preprints/socarxiv/5n3y8/.

[15] R Core Team. R: A Language and Environment for Statistical Computing. Vienna, Austria; 2019. Available from: https://www.R-project.org/.

[16] Plummer M, et al. JAGS: A program for analysis of Bayesian graphical models using Gibbs sampling. In: Proceedings of the 3rd international workshop on distributed statistical computing. vol. 124. Vienna, Austria.; 2003. p. 10.

[17] Su YS, Yajima M. R2jags: Using R to Run 'JAGS'; 2015. R package version 0.5-7. Available from: https://CRAN.R-project.org/package=R2jags.

[18] Plummer M. rjags: Bayesian Graphical Models using MCMC; 2018. R package version 4-8. Available from: https://CRAN.R-project.org/package=rjags.

[19] Gelman A, Rubin DB, et al. Inference from iterative simulation using multiple sequences. Statistical science. 1992;7(4):457–472.

[20] Plummer M, Best N, Cowles K, Vines K. CODA: Convergence Diagnosis and Output Analysis for MCMC. R News. 2006;6(1):7–11. Available from: https://journal.r-project.org/archive/.

[21] Alkema L, Wong MB, Seah PR. Monitoring progress towards Millennium Development Goal 4: A call for improved validation of under-five mortality rate estimates. Statistics, Politics and Policy. 2012;3(2).

[22] Alkema L, Chao F, You D, Pedersen J, Sawyer CC. National, regional, and global sex ratios of infant, child, and under-5 mortality and identification of countries with outlying ratios: a systematic assessment. The Lancet Global Health. 2014;2(9):e521–e530.

[23] Chao F, You D, Pedersen J, Hug L, Alkema L. National and regional under-5 mortality rate by economic status for low-income and middle-income countries: a systematic assessment. The Lancet Global Health. 2018;6(5):e535–e547.

[24] Chao F, You D, Pedersen J, Hug L, Alkema L. Web appendix National and regional under-5 mortality rate by economic status for low-income and middle-income countries: a systematic assessment. 2018. DOI: 10.6084/m9.figshare.12442244. Available at https://ars.els-cdn.com/content/image/1-s2.0-S2214109X18300597-mmc1.pdf.
